# Combating Fear of Missing Out (FoMO) on Social Media: The FoMO-R Method

**DOI:** 10.3390/ijerph17176128

**Published:** 2020-08-23

**Authors:** Aarif Alutaybi, Dena Al-Thani, John McAlaney, Raian Ali

**Affiliations:** 1 Faculty of Science and Technology, Bournemouth University, Fern Barrow, Poole BH12 5BB, UK; jmclaney@bournemouth.ac.uk; 2 College of Science and Engineering, Hamad Bin Khalifa University, Doha PO Box 34110, Qatar; dalthni@hbku.edu.qa (D.A.-T.); raali2@hbku.edu.qa (R.A.)

**Keywords:** digital addiction, digital wellbeing, fear of missing out, FoMO

## Abstract

*Background:* The fear of missing out (FoMO) on social media refers to the apprehension that online content and interactions from others are unseen and reacted to in a timely fashion. FoMO can become problematic, leading to anxiety, interrupted sleep, lack of concentration and dependence on social media to generate gratification. The literature has mainly focused on understanding the FoMO experience, factors contributing to it and its consequences. *Method*: In this paper, we build on previous research and develop a FoMO Reduction (FoMO-R) approach that embraces technical elements such as autoreply, filtering, status, education on how FoMO occurs and skills on how to deal with it; e.g., self-talk and checklists. We evaluate the method through focus groups and a diary study involving 30 participants who self-declared to experience FoMO regularly. *Results:* The results show that the method was accepted by the participants and helped them to manage their FoMO. They also show that a set of extra functionalities in social media design is needed so that users can manage FoMO more effectively. *Conclusion:* FoMO can be reduced through socio-technical approaches, joining both social and technical skills, and literacy on how social media are designed and how social interactions should happen on them.

## 1. Introduction

In recent years, social media has reshaped the way individuals interact. On the one hand, it has provided new means to develop relationships and remain socially related, and has facilitated more reciprocal and continuous interactions among individuals, regardless of time and location. On the other hand, such novel social communication method has triggered a new range of negative consequences where virtual communities operate in different modalities in comparison to traditional communication and where a new set of interaction norms are perceived to exist by the individuals [1]. One such negative experience is the fear of missing out (FoMO). This typically refers to the preoccupation of users of social media with lost opportunities when they are offline or unable or unwilling to connect and communicate with others to the extent they wish [2]. Researchers have recently begun to explore the negative consequences of FoMO that are faced by social media users. FoMO is a main type of problematic attachment to social media, and is associated with a range of negative life experiences and feelings, such as a lack of sleep, reduced life competency, emotional tension, negative effects on physical well-being, anxiety and a lack of emotional control [3,4]. Despite indicators of the effect of FoMO on users’ well-being, guidance and tools that allow people to manage it are still not available.

To date, most of the empirical research concerning FoMO has been conducted on social science and psychological grounds. For example, in [5], a correlation between motives for social media use and social media addiction was found. The definition of digital addiction in the literature was focused on usage styles and harm associated with them [6]. The role of social media in triggering problematic usage styles and also in correcting them is still an under-researched topic [7]. Our literature search returned no methods proposed to combat FoMO. In the general area of internet addiction and gaming disorder, current approaches still need further evidence and are based on counselling and interventions, e.g., cognitive behavioural therapy, prevention action-research, and family therapy, besides pharmacological therapies [8]. Existing approaches overlook the role of social network design features in triggering the problem and, at the same time, their potential to play a role in solving it. In addition, literacy about online usage and its relation to the design and factors such as the social pressure it facilitates, is yet to be studied in terms of its impact on creating resilience to FoMO. In this paper, we argue that social media can play a part in triggering FoMO and can be designed to provide preventative measures. Solutions shall be socio-technical, and design teams shall be inter-disciplinary, involving members with skills in software engineering, interactive systems, social psychology and behavioural change.

Previous research on FoMO has built on retrospective data collection methods. Abel, et al. [9] used a survey in order to explore the measurement of FoMO. In [10], the relationship between the utilization of social networks, FoMO, self-promoting behaviour, online vulnerability, and psychological wellbeing were investigated through extensive online surveys. In [11], an online survey was used to explain problematic smartphone use in relation to FoMO. Przybylski, et al. [12] conducted three studies to understand FoMO, and they used online survey and interviews. A paper-based survey was used in [13] to examine the relationship of adolescents’ FoMO with adolescents’ perceived stress related to the use of Facebook. An online survey was used to investigate the association between social networks use, FoMO and a broad range of clinically relevant health outcomes [14]. A survey and focus group were used to understand the experience of FoMO when people study abroad [15]. A focus group was used to uncover peoples’ negative psychological and relational experiences associated with social networks such as FoMO [16]. Alt [17] used a survey to investigate the link between FoMO, social network sites engagement and three motivational constructs. This introduces limitations due to the possibility of recall bias and questions about ecological validity. To mitigate such shortcomings, Alutaybi, et al. [2] explored the lived experience of individuals who suffered FoMO and sought to understand the phenomena in a naturalistic setting. They utilised a diary study design as a data collection method in the exploration phase. These studies developed five primary contexts of use in which FoMO occurs and the specific fears in each context [2]. The same authors explored features of social media that could facilitate FoMO and, in addition, the existing and future social media features that alleviate FoMO [18,19].

In this paper, we develop a FoMO Reduction method (FoMO-R). The method associates between the context of use, fears and technical countermeasures. It also provides educational material that explains how FoMO occurs and how it can be dealt with; e.g., through checklists, self-talk and expectation management. It is hoped that this might build the users’ digital resilience and help them to prevent and deal with certain kinds of FoMO. We used the results of previous research in [2,18,19] to devise the main components of FoMO-R and added to them, particularly at the level of social countermeasures. We evaluate the potential of the method empirically through focus groups and a diary study involving 30 participants. A positive change in managing FoMO was reported. Participants also reported a positive experience with the method steps and material. The results also show that more social media features are needed to act as technical FoMO countermeasures. Future social media designs can introduce them to aid users with their digital wellbeing. The paper is structured as follows: in Section 2, we present the theoretical underpinnings of the FoMO-R method. In Section 3, we explain the FoMO-R stages and strategies. In Section 4, we describe the FoMO-R evaluation study. In Section 5, we discuss the paper, and in Section 6, we conclude it and present future research directions.

## 2. FoMO Reduction Method (FoMO-R): Theoretical Underpinnings

In this section, we discuss the theories that informed the design of the FoMO-R method, based on both theories that relate to the reasons an individual experiences FoMO and the theories that have been used to bring about behaviour change and improvements in wellbeing in other domains.

### 2.1. Types of FoMO and Their Technical Countermeasures

Previous research [2,18,19] has identified five primary contexts of use where FoMO occurs, the fears associated with them and the potential technical countermeasures. The findings are summarised in Table 1. FoMO can happen when others do not interact as expected, when unable to interact or connect as wished, when unwilling to engage in social interaction when having to, or feeling a need to engage in continuous untimed interactions and when an online social gathering is expected. Fears associated with these contexts include the fears of missing the ability to be popular and interesting to others, the fear of not getting the right interpretation of some social interaction, the fear to deal with different social networks at one time, and the fear of missing temporarily available information and timely interactions. Countermeasures for these fears include filtering, autoreply, setting status and recap. These are software-assisted countermeasures, and we recognise the need for a human counterpart. Later in this paper, we propose skills and techniques in expectation management, empowered refusal and self-regulations to be used together with the technical countermeasures.

### 2.2. Self-Control Theory

Self-control has been defined as the effort that is exerted to regulate the self by the self. Self-control occurs when individuals try to alter the way in which they would otherwise think, feel or behave [20]. In the absence of self-control, individuals will carry out a particular desired behaviour; that is, they fail to delay gratification [21]. This may occur when using social media when individuals are unable to interact with or connect to social media as they would like, e.g., during a meeting. They may still check impulsively due to their limited ability to postpone gratification. The reason behind this may be FoMO, in which people fear being ostracised from interaction with their friends or missing an opportunity [22]. This relates to classification 2 of FoMO, as shown in Table 1.

### 2.3. Anxiety Management

Anxiety is a significant part of the FoMO definition in the literature; e.g., “the fears, worries and anxiety people may have about being in (or out of) touch with events, experiences and conversations happening across their extended social circles” [23]. Given the underlying fear component of FoMO, anxiety relates to each of the five classifications of FoMO and is especially relevant to the first, second and fourth classifications, where there is an element of the individual experiencing a loss of control (Table 1). Anxiety management can be an effective and safe technique as an alternative to anxiolytic drugs for dealing with anxiety disorders [24]. It is an active self-help remedy relying on the vicious circle model of anxiety. Cognitive reappraisal of anxiety [25] has been used to help people who have digital addiction, and its effectiveness has been proven by people who have learned to recognise their digital addiction and to manage it [25]. Consequently, it can be potentially useful to prepare people to identify FoMO triggers through their thoughts and feelings. Cognitive reappraisal of anxiety will be used in the planning stage of FoMO-R.

### 2.4. Compulsive Behaviour

FoMO motivates individuals to continually check their social media accounts in order to remain strongly connected through their social relationships and also to avoid the worry of not being up to date with opportunities to gain as well as losses to avoid. It can lead to compulsive behaviour, and people may fail in resisting the urge to check their social media or stop being preoccupied about them. Compulsive behaviour is related to addiction, as an “addiction is a persistent, compulsive dependence on a behaviour or substance” [26]. The definition goes on to state that there are two types of addiction: “substance addiction” and “process addiction”. Substance addiction is an addiction to substances such as cocaine or alcohol. Process addictions comprise compulsive behaviours that do not depend on a consumable substance, such as gambling [27]. In this case, it is the actual process of doing something that may make individuals addicted. According to this definition, FoMO meets certain symptoms of process addiction, mainly the salience of social media, the withdrawal symptoms and also the conflict with other priorities as well as mood modification. Compulsive behaviour is a kind of anxiety disorder in which intrusive and subjectively uncontrollable thoughts are the main features. It relates most closely to the second and fifth classifications of FoMO shown in Table 1. Compulsive behaviour can be regulated using techniques such as distraction and reappraisal [28]. Distraction avoidance and reappraisal techniques will also be used in FoMO-R.

### 2.5. Uncertainty Avoidance

Uncertainty avoidance is defined as the extent to which the members of a culture feel threatened by uncertain or unknown situations and is “related to anxiety, [the] need for security and dependence upon experts” [29]. It is also the extent to which an organisation or group depends on social norms or rules to alleviate the unexpected in future events. People with high uncertainty avoidance are active, aggressive, emotional and compulsive [30]. Thus, if a situation does not fit their perception or expectation of what is reasonable, they will create a degree of uncertainty that leads them to look for the cause. This can be seen in interactions on social media; when an experience does not meet an individual’s expectations for interaction, they may seek answers as to why others do not interact with them, why interaction is lacking, or the style of interaction is insufficiently comprehensive. Hence, they may experience one or more kinds of FoMO, such as fear of missing the ability to be popular, fear of missing the ability to be interesting or fear of missing the ability to get the right interpretation. Such FoMO may lead them towards unhealthy behaviours, for example, cyberstalking or compulsive behaviours aimed at avoiding uncertainty. Interactions on certain social media among people should be obvious and declared to avoid any negative consequences, i.e., FoMO. As such, uncertainty avoidance is connected to all five classifications of FoMO shown in Table 1.

### 2.6. Attribution Theory

The desire to explain the behaviour of others is a reflection of the fact that humans are a fundamentally social species whose survival and success are determined in part by how well they can predict and navigate their social world. Kelley [31] noted that humans are influenced by the real, implied or imagined presence of others. With the rise of social media, the ‘presence’ of others has become ubiquitous. This has brought about a fundamental change in the locus of control in how people interact with their social group. Before social media, individuals may have attributed the frequency or lack of their interactions with their social group to the constraints of not being physically together. As such, there would be extended periods of time when individuals would not easily be able to communicate with each other. Importantly, however, they would be aware that those within their social group had the same understanding of the situation. Since the development of smart devices and social media, it is now almost always possible for people to communicate and interact with their social group. Thus, the locus of control for social communication and interaction has altered from partly externally determined to largely internally determined. However, the increased social interaction opportunities and the easy access to social media may cause individuals to experience FoMO, which is a preoccupation with what is occurring online and how others are reacting, either positively or negatively, to someone’s online presence and interactions. This relates to all five of the classifications of FoMO shown in Table 1, especially the first classification.

### 2.7. Resilience and FoMO

Resilience is often described as “a pattern of adaptive functioning in the presence of life adversities” [32,33]. Another common definition describes it as “the process of, capacity for, or outcome of successful adaptation despite challenging or threatening circumstances” [32]. In the past few decades, the definition has become more consistent [34], but there is a specific distinction between considering resilience as an outcome or as a non-static developmental process. Hence, the concept is difficult to define because it is not one simple construct but a combination of biological, psychological and sociological processes and interactions. This indicates the importance of understanding that resilience is a process of changing risk and protective factors that affect how people adapt in different contexts.

The common themes that arise from the definition of resilience is risk, which is any unwanted circumstance that people face when taking action [35], and it aids the process by which they maintain adaptive functioning. Resilience consists of precautionary factors that “moderate against the influence of a stressful situation so that people are able to adapt more successfully than they would have, had the protective factor not been present” [36]. Thus, resilience is contingent on a stress-moderating or buffering process. There is controversy about the notion of resilience and the standard values that support what is considered ‘good’ functioning [37,38].

New definitions of resilience have been developed by researchers to take into consideration how young individuals navigate online and the risks they encounter. Online resilience is defined as “being able to deal with a negative experience online; i.e., not remaining passive but displaying problem-solving coping strategies in order to protect oneself from future harm” [39]. Several elements have been shown to help young individuals to successfully navigate and overcome online experiences, including confiding in an adult [40], talking about the experience with someone [41], being digitally literate [39], having parents who facilitate their children’s online media environment [42], and having supportive peers [43].

Based on the definition of online resilience, FoMO is one of those negative online experiences that is associated with negative consequences [11,13,15,17]. The broader issue of digital addiction, which can be an advanced stage of FoMO, is associate with depression and negative emotions [14], eating disorders [13], low life competency [11], emotional tensions [44,45], negative effects on physical well-being [17], anxiety [46], emotional control [47], and insufficient sleep [48]. FoMO-R is aimed at building resilience in making people who use social media aware of how FoMO happens and how it can be managed, whether in applying technical countermeasures or social ones. As such, resilience is a factor that pertains to the FoMO-R method for all five classifications of FoMO shown in Table 1.

### 2.8. Transtheoretical Model

The transtheoretical model is one of the prominent models in the field of behaviour change research. It is widely used by many researchers and practitioners. The model proposes that behaviour change goes through five milestones (pre-contemplation, contemplation, preparation, action and maintenance) and is an attempt to combine ten core processes of behavioural change including consciousness-raising, counter-conditioning and stimulus control, which are mapped to the stages of the model [49]. Several processes belong to more than one stage. This model will be taken as a starting point for the stages and processes for the FoMO-R method.

### 2.9. Self-Talk

People may find it difficult to say ‘no’ to temptation, but it is an important skill that we all need to protect our health and well-being [50]. Distractions and temptations can often affect an individual’s desired goals; for instance, social media can, to some extent, motivate procrastination, even when the individual wishes to regulate their usage [51]. Self-talk is a tool that can be used to frame an individual’s resistance to temptation and to motivate goal-directed behaviour. Additionally, because self-talk affects the mental representation of one’s selections, a refusal framed to signify a sense of empowerment and control has the potential to be effective in self-regulation [50]. The use of ‘do not’ can be effective when resisting temptation and motivating goal-directed behaviour because it provides a sense of psychological empowerment and also conveys force and determination, and is therefore final and not to open to discussion [52,53]. Self-talk words are helpful in the mitigation of FoMO; for example, one might say “I do not need to respond swiftly” instead of “I am unable to respond swiftly.” 

The FoMO-R user will be told that each time they experience FoMO, they should tell themselves “I don’t do X.” Additionally, they will be asked to rehearse the strategy. For instance, when unable to interact or connect as they wish, they should say “I do not have to check all my friends’ updates on social media,” “I do not need to know everything people say about me” or “I chose not to check each notification immediately.”

### 2.10. Expectation Management 

An expectation is a strong belief that something will happen. It can be formed based on attitudes, values or norms [54,55]. In terms of communication and interaction on social media, individuals may form expectations of online interactions based on previous interactions with others, such as receiving responses immediately or receiving ‘Likes’ or ‘Retweets’. Additionally, individuals attempt to avoid being perceived as alien and aim to maintain a positive self-concept by conforming to the norms and improving reciprocity behaviour online [56]. This may drive them to be more active on social media in order to avoid experiencing FoMO that might be the fear of missing the ability to defend their popularity or fear of missing the ability to be interesting. For this reason, expectation management is included in our proposed FoMO-R method. Individuals need to learn to manage their expectations when they are involved in online interactions. This can be achieved by utilising the cognitive reappraisal method [25]. For example, individuals can manage their FoMO by posting on social media without expecting interaction from others. Similarly, they can clarify to others that they may not reciprocate interaction. Another example of expectation management which helps users to avoid feelings of ostracisation by others on social media is to make clear to their social media contacts their current situation, e.g., being busy preparing for an exam or having limited time.

### 2.11. Enhancing Self-Esteem

Self-esteem is an individual’s subjective evaluation of their own worth. If an individual feels unpopular because they have not achieved the number of ‘Likes’ for a post they expected, they may experience stress and anxiety [28]. They may link that to missing out on previous interactions with others and that they could not interpret it well. Hence, their FoMO increases due to the desire to increase their knowledge of their social circle well. To combat FoMO, it is necessary to enhance self-esteem to mitigate a certain kind of FoMO. This can be achieved by practicing a change in attitude. Individuals might say: “my popularity is not measured by being active on social media and immediately responding to messages” or “I am not the only one who does not receive the interactions they expect.”

## 3. FoMO-R Stages and Strategies

FoMO-R is built upon steps inspired by the transtheoretical model [57], and incorporates work around the theoretical underpinnings of the causes of FoMO and the possible techniques that may be used to reduce FoMO. Although we have identified five classifications of FoMO, it is acknowledged that any behaviour can be multi-faceted and complicated and that it would be simplistic to assume that each individual may only experience one type of FoMO. FoMO types are by nature context-dependent, e.g., some happen when one is unable to communicate due to the technical or social context, and others happen when one is not receiving interactions from others and unable to interpret the reason. As such, our FoMO-R method is intended to be inclusive to all individuals who may have different FoMO experiences. They can choose more than one kind of FoMO they typically encounter and more than one countermeasure to combat each of them, depending on their capabilities and preferences.

The transtheoretical model involves progression through four stages of change (preparation, planning, action and review) and requires the raising of consciousness to achieve a change in behaviour. FoMO-R was designed in a user-friendly style that included a self-help guide and four different booklets. The material can be found in the supplementary material.

A self-help guide booklet provides information regarding FoMO and how it can occur on social media. In addition, the self-help guide introduces FoMO-R and how it can be used by those seeking help. 

The self-rating booklet (Booklet 1) introduces the classifications of FoMO to people who typically experience it when using social media.

The FoMO countermeasures booklet (Booklet 2) presents people with a variety of countermeasures that can be used to manage different kinds of FoMO.

The relapse prevention booklet (Booklet 3) gives those seeking help a set of skills and techniques designed to help them to cope with the problem of relapse.

The empowered refusal booklet (Booklet 4) provides those seeking help with further support to manage their FoMO in the event that the countermeasures selected from Booklet 2 fail. 

The stages of FoMO-R are summarised in Table 2. We elaborate on each stage in the next sections.

### 3.1. Preparation Stage

The purpose of the preparation stage is to raise self-awareness of FoMO, and also to provide help-seekers with a greater understanding of the various types of FoMO. This is the stage in which help-seekers intend to manage their FoMO in relation to social media. They may be involved in this stage because they are unaware exactly how FoMO happens and of its consequences, or they may have attempted to manage it but have low self-efficacy, which means that they fail to enact the required change.

Raising awareness is achieved by introducing the classifications of FoMO to help-seekers and asking them to select the FoMO type(s) that they typically experience by referring to the self-rating booklet. Booklet 1 is based on the results from previous work that was conducted by Alutaybi, et al. [2]. The help-seeker is asked to tick the symptoms that they feel when they experience a certain FoMO situation and is given a self-rating booklet to diagnose their FoMO based on their daily usage. Table 3 shows a sample of self-rating, and further information about the booklet with the user version can be found in the supplementary material. The aim of this stage is to make individuals recognize their FoMO and how it arises on social media, and also to prepare them for the next stage, the planning stage.

### 3.2. Planning Stage

The aim of this stage is to assist help-seekers in planning for their goal of managing FoMO by providing them with a set of suggested countermeasures. This includes learning:

*Technical countermeasures such as auto-reply and set status*

*Socio countermeasures such as self-talk, managing the expectations of others, and self-control.*

*Relapse prevention strategies, which will prevent the help-seeker from reverting to old habits.*

This stage is based on the cognitive reappraisal of anxiety and social media use method [25]. Here, the help-seeker will learn about their daily social media use, cognitions involved in this use, and anxiety. Cognitive distortions are determined in the first stage, and the help-seeker comes to realise that such distortions facilitate the experience of FoMO and the excessive use of the internet; e.g., “I have to answer my friends immediately, otherwise they will not forgive me”; “If my friends don’t give ‘likes’ on my posts or my photos, it is a signal that they don’t like me or that I did something wrong”; and “If I am unable to connect to social media, I will miss important or valuable things because the best things are on social media.” All thoughts related to anxiety and social media use were extracted over two studies and explained in [2]. The authors in [2] also determined five classifications of FoMO. It appears that when users of social media do not receive the interactions they expect, they may experience different fears. The reason why they did not receive the expected communication could vary, e.g., the recipient did not find the content attractive, they may have been unavailable to interact or they may have been unable to understand the language or symbols used. Thus, if the senders were aware of these reasons, they prepare for them proactively to avoid facing FoMO later. Therefore, a pre-posting thinking model has been proposed that can help people to manage their FoMO before it arises. Pre-posting thinking is a basic strategy that can help individuals to post on social media without experiencing negative feelings. We propose VFFT (derived from Value, Fitness, Format and Time) guidelines to help individuals think before posting on social media:

- Value: Individuals should ask themselves whether the quality of, or idea behind, the post is important and will benefit people on social media.
- Fitness: Does the content of the post fit the interests of those targeted?
- Format: Individuals should check the representation and language used in the post.
- Time: Users should be aware of the availability of others on social media. They should bear in mind the time zones of contacts/followers in other countries.

When VFFT is followed, individuals have to consider the following expectations:

- Expect few interactions
- Expect no interactions
- Expect no immediate response
- Expect that not all your contacts are interested
- Expect others are on leave
- Expect others may need time to process
- Expect that the algorithm may not show the post to the intended recipient
- Expect that others were unable to connect to the internet
- Recognise that if they do not receive a response from someone who is online, they may be involved in an urgent or business conversation
- Recognise that if they do not receive a response from someone who is online, they may not be prepared to answer

In order to support FoMO-R, VFFT is combined with a variety of methods that are intended to educate the help-seeker about reducing the feeling of FoMO. Help-seekers are asked to select suitable technical and/or socio countermeasure(s) from the FoMO-R countermeasures booklet (Booklet 2) for each type of FoMO that was selected from the self-rating booklet (Booklet 1) in the previous phase. Booklet 3 helps the user to find out about relapse prevention and supports their countermeasures. Table 4 and Table 5 show samples of FoMO-R countermeasures and relapse prevention strategies, respectively, and further information about booklets with user version can be found in the supplementary material. After this stage is achieved, the help seeker will go to the next stage, the action stage.

### 3.3. Action Stage

Help-seekers can manage their FoMO and regulate their usage style on social media. The help-seekers are asked to spend a period of time, typically one week, practicing and rehearsing the techniques they have selected from the previous stage, the planning stage. In this stage, the help-seeker is reminded by doing activities that were declared by them in the planning stage in order to prevent a relapse.

### 3.4. Assessment Stage

Help-seeker is asked to assess their own satisfaction with using the selected countermeasure(s). This assessment is intended to assess the extent to which using the selected countermeasures made them feel psychologically empowered. The assessment works as follows:

- For each countermeasure that help-seekers selected for each of the FoMO types they have, they indicate whether it was useful for them.
- If they found at least one useful countermeasure for each of their FoMO types, they move on to Stage 4, the review stage.
- If they have one or more of the FoMO types without any useful countermeasures, they move on to the next step, the empowerment step.

### 3.5. Empowerment

Help-seekers find further support. To do so, they determine the challenges that may make it harder for them to manage their FoMO by answering the following questions:

- Is peer pressure the cause?
- Do you put the needs of others above your own?
- Are there technical issues?

**Figure 1:**
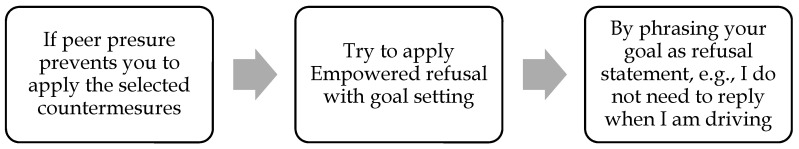
Sample of empowered refusal and self-talk booklet (Booklet 4).

After having thought about these questions, they have a choice to select other countermeasures from the FoMO countermeasure document (Booklet 2), or following the instructions in Booklet 4, return to the application stage and repeat. Figure 1 shows a sample of Booklet 4 and further information about this booklet with the user version can be found in the supplementary material of this paper and in [58]. 

If they run out of countermeasures and are unable to cope with their FoMO, they move on to the next stage, the review stage.

### 3.6. Review Stage

In this stage, help-seekers review what they did in the previous stages to see whether they have managed their FoMO. This stage works by asking the help-seekers to answer the questions: (1) What happened? (2) What has been improved? and (3) Did you manage your FoMO? If they managed their FoMO, they repeat the first stage (the preparation stage) to see whether there are any other FoMO types that apply to them. If they do not select any additional types of FoMO, the FoMO-R process is ended. However, if they failed to manage their FoMO, they are asked to assure themselves that they selected all FoMO types that applied to them and that they maintained an appropriate focus on the selected countermeasures and the relapse prevention technique. For those who saw no improvement, this may be indicative of comorbidity.

## 4. FoMO-R Evaluation

Following on from the previous section where FoMO-R was introduced, this section reports on how we obtained evidence of the potential of FoMO-R to help to reduce FoMO and its other qualities, such as ease and readability. In this section, we report on the design of the FoMO-R evaluation process, the findings that emerged and, finally, the conclusions are drawn.

### 4.1. Design of the Evaluation Study

This section sets out the procedures put in place in relation to participant recruitment and data collection. For the complete set of materials used in the evaluation study, please refer to [58]. All the study went through the ethics approval process of the authors’ institution. The evaluation process comprised three steps, and there are a number of activities that have to be undertaken in each step (see Table 6):

#### 4.1.1. Sample Recruitment

Thirty participants were recruited by an open call being issued through a student forum, where individuals could self-nominate to participate. All participants self-identified that they suffer from FoMO, in relation to their social media usage. Participants received a consent form and details outlining the purpose of the study through email in advance in order to give them adequate time to peruse these documents and seek further information, as required.

#### 4.1.2. Focus Group

The first phase, i.e., before the use of FoMO-R, consisted of convening three focus group sessions, with a maximum of ten participants assigned to each. The focus group sessions were specifically designed to give participants an opportunity to immerse themselves in the issue, discuss their opinions about how FoMO occurs on social media and be instructed on how to use the FoMO-R. In addition, participant demographics and FoMO experience data were collected by administering a survey. Each participant was given FoMO-R materials that can be found in the supplementary materials of this paper. Ten participants were allocated to each of the three focus groups. Ages ranged between 18 and 42 years.

#### 4.1.3. Diary Study

The second phase, i.e., during the use of FoMO-R, consisted of a diary study, comprising the 30 participants who had already participated in the focus group sessions. As part of this study, participants were requested to apply the FoMO-R over ten days. The diary studies approach was also chosen as it minimises recall bias and provides the opportunity to gather in-play data, which are more expressive and contextualised [59]. During the diary study phase, participants were requested to complete the treatment questionnaire concerning continued programme participation TSRQ [60] after three days. They were also asked whether they had encountered issues or difficulties when they were applying the FoMO-R using the diary template. The aim of this question was to take participants’ timely feedback into consideration when conducting the analysis. Participants were provided with a hard copy document on which to write their diary entries, and they were also reminded to record this information through the use of text messages.

#### 4.1.4. Questionnaire

The third phase, i.e., after the use of FoMO-R, consisted of administering a paper questionnaire, which comprised three parts. The first part of the questionnaire focused on participant FoMO experiences. The second section included the e-Therapy Attitudes and Process Questionnaire (e-TAP), which is based on the Theory of Planned Behaviour. The final part contained a combination of open-ended and close-ended questions (e.g., ratings). The open-ended questions were used to minimise the risk of missing important information and to allow participants to feel free to add information they felt was relevant to the FoMO-R evaluation process. Closed questions were used to stimulate or trigger participant thought processes while also ensuring that they had to invest less effort in completing the questionnaire. These questions focused on the usefulness, coverage and clarity of the FOMO-R. Participants who completed the diary study undertook the questionnaire, having used the FoMO-R. The questionnaire was employed to assess the extent to which participants understood the purpose that the FoMO-R is serving. This was achieved by undertaking a comparative analysis of participant answers gathered through the focus group sessions with their responses elicited through the questionnaire.

#### 4.1.5. Analysis

Data analysis was carried out in order to address the research questions set out earlier. Essentially, its primary objective was to evaluate the proposed method (i.e., the FoMO-R), by establishing whether it is sufficiently comprehensive. This included considering factors such as its awareness-raising capability regarding FoMO types and how it can be managed, and determining if its supplementary materials (self-rating and countermeasure education documents) provide sufficient information about how FoMO occurs on social media and how it can be managed.

The returned evaluation forms were analysed, where the responses were cleaned up and irrelevant/inconsistent answers were excluded. Descriptive analysis of the quantitative aspects of the survey was conducted in order to describe the data. In addition, a series of paired sample t-tests were performed to test whether any difference in FoMO experiences occurred before and after using the FoMO-R. Paired sample t-tests were done on the total score within each context, instead of on individual questions. Qualitative analysis was also applied to the open-ended questions contained in the survey, which comprised coding the responses, as well as identifying patterns and trends, which were subsequently classified into various categories. The quantitative and qualitative findings were compiled, and they were then reported on collectively.

### 4.2. Findings

The findings were structured in line with the question format adopted in the evaluation. In addition, the diary study and open-ended questions, as the qualitative components of the evaluation, were reported on in conjunction with the quantitative aspect. Quotes will be differentiated in the text using italics.

#### 4.2.1. Sample

Thirty participants took part in this study. All participants stated that they were keen to learn more about how FoMO manifests and how it can be managed on social media. Therefore, fifteen men and fifteen women (*n* = 30), ranging in age from 18 to 42 years (M = 26.37, SD = 5.89), participated in this study.

#### 4.2.2. FoMO-R Coverage

**Figure 2:**
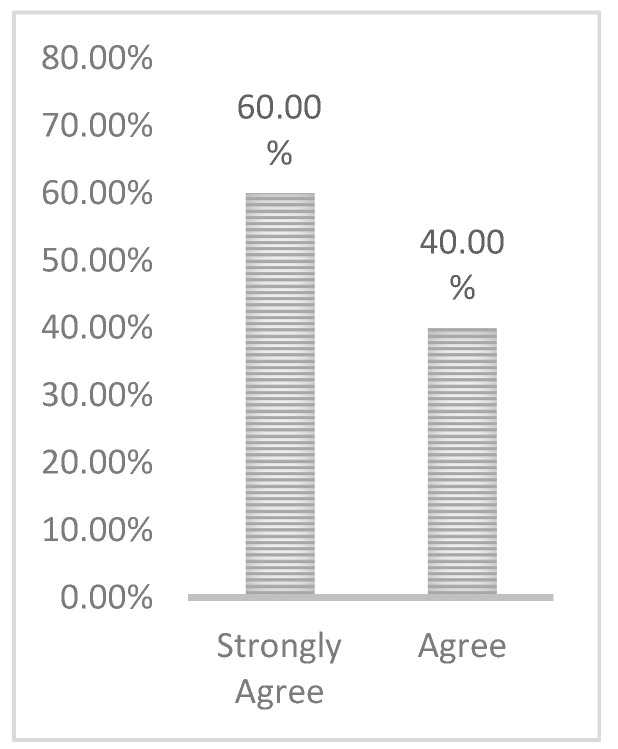
FOMO-R coverage.

The questions regarding coverage are in Appendix A. The response to the question regarding FoMO-R coverage is shown in Figure 2. It can be seen that the largest proportion of the sample strongly agreed that they had received sufficient information on how to use the FoMO-R, with 60% strongly agreeing with this statement. It can also be noted that no one expressed a negative attitude toward the completeness of the FoMO-R information provided as a self-help guide to respondents. 

One participant commented on the coverage of FoMO-R, stating that: “The booklets provide step by step plans for identifying, treating and preventing FoMO in users in an effective way”. Another remarked that, “The whole process was explained sufficiently in the self-help guide with the use of diagrams and figures”.

**Figure 3:**
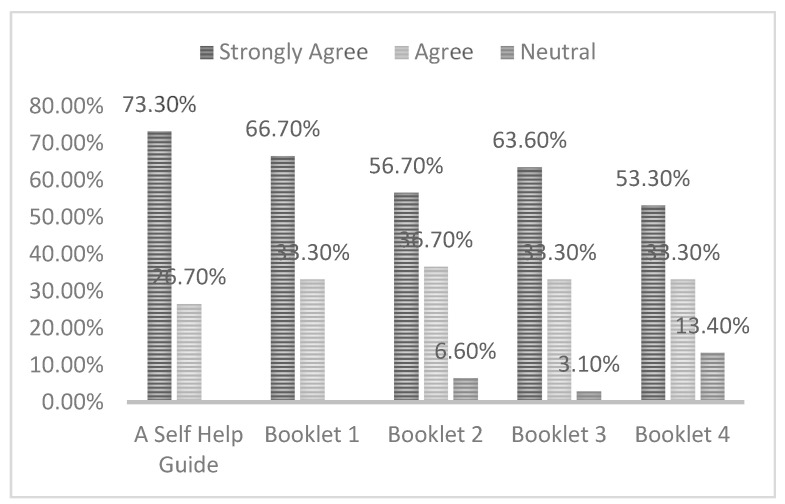
Supplementary materials coverage.

Moreover, Figure 3 shows the response to questions regarding the coverage of the FoMO-R materials used, namely, the self-help guide and Booklets 1–4; the following sections explain the responses received for each booklet separately.

In the question relating to the coverage of the self-help guide (which provides information on how FoMO occurs on social media), most of the participants displayed a strong positive attitude toward it. They stated that it provided sufficient information about FoMO in relation to social media use, with 73.3% strongly agreeing with this statement. A further 26.7% of participants displayed a positive attitude by agreeing with the statement. It can also be noted that no participant expressed either a negative or strongly negative reaction, or indeed a neutral opinion.

Additionally, a question was included relating to the coverage of Booklet 1, which provides statistics regarding the large numbers of FoMO that exist within different contexts. In response to this question, it can be noted that a large proportion of the participants (66.7%) strongly agreed that the information provided in Booklet 1 was complete, while the remaining one-third (33.3%) agreed with this statement.

Most participants provided positive comments regarding the coverage of Booklet 1. One such example is captured in the comment that, “*Booklet 1 was helpful for us and make me aware of what types of FoMOs occur on social media”*. Another participant commented on the colour used for this booklet, stating, “*The different kinds of FoMOs were divided into sections and the colours helped to place the different FoMOs into groups. Lots of them you experience yourself so it’s easy to remember”.*

Booklet 2 outlines a variety of countermeasures that exist, which are either technical or social, and they can help people in managing the different forms of FoMO. With regard to the question on the coverage of Booklet 2, analysis has shown that most participants strongly agreed that it contained sufficient countermeasures combating different types of FoMO, with 56.7% strongly agreeing with this statement. A further 36.7% reacted positively to the booklet, by agreeing that the key information was included. Finally, a neutral opinion was held in relation to this question by 6.6% of the participants. 

Overall, the comments were positive in relation to the coverage in Booklet 2. One of the comments focused on the detailed description provided of each countermeasure, with one participant noting that, *“Each countermeasure has sufficient description that helps us to apply it and to identify the best one”.* Another participant indicated that, *“There are plenty of countermeasures provided that help them in managing their FoMO”.*

Furthermore, descriptive analysis of the question on the coverage of Booklet 3 showed that most participants strongly agreed that the relapse prevention booklet outlined an adequate number of techniques aimed at preventing the recurrence of relapse, with 63.6% strongly agreeing with this statement. In addition, a further 33.3% of participants expressed a positive reaction toward it. A neutral opinion regarding the completeness of Booklet 3 was held by 3.1% of participants. It can be seen that participants who expressed a neutral opinion they might not use Booklet 3, as it is for relapse prevention, while the countermeasures for FoMO that are in Booklet 2 can be enough. This could be inferred from one of the participant’s comments: “*I do not use Booklet 3 because I found countermeasures are useful to me*”. The comments provided further reinforced that participants supported the details contained in Booklet 3, with one of them describing it as “*a reminder for me about comprehensive activities and techniques that help me to prevent the occurrence of relapse through the use of countermeasures”.*

Booklet 4 sets out further countermeasures which can help people if those suggested in Booklet 2 are not sufficiently useful for them. Analysis of the data emerging from the question regarding the coverage of Booklet 4 revealed that 53.3% of participants strongly agreed that the booklet was useful, while a further 33.3% agreed with this statement. A neutral opinion was held by 13.4% of participants, and whereby it can be seen that in all subsequent graphs the total percentage of neutral opinions expressed never exceeded 13.4%. This higher neutral rating might be reflective of the fact that these participants did not use Booklet 4.

Moreover, the comments provided by participants supported the views held regarding the coverage of the issue in Booklet 4, where one participant stated that, “*There was lots of information given and it was easy to understand when we could not find another useful countermeasure”.* Another noted that, *“Booklet 4 empowerment methods explain and identify very clearly and concisely ways to help the user overcome FoMO and empower themselves”.*

#### 4.2.3. FoMO-R Clarity 

**Figure 4:**
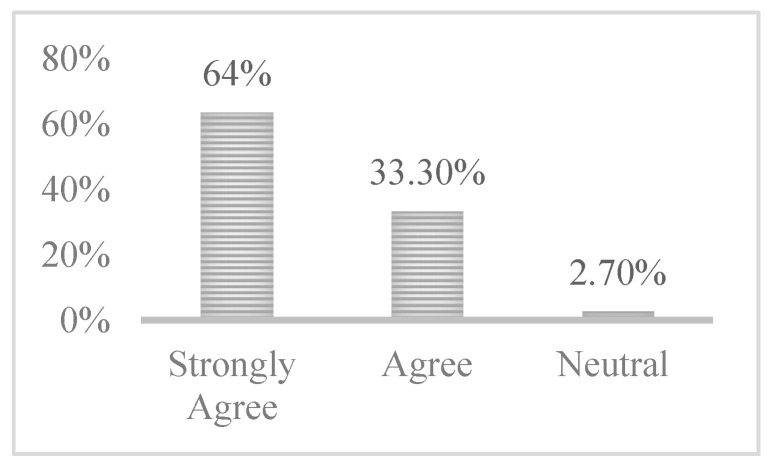
FoMO-R clarity.

One of the aspects considered in this study was the level of clarity surrounding the FoMO-R, in terms of how clear and easily understandable it is for help seekers. In response to the question regarding this aspect, the results, shown in Figure 4, demonstrate that the largest proportion of participants strongly agreed that the FoMO-R was not difficult to understand and was explained in a clear manner, with 70% strongly agreeing. It can also be noted that no participant displayed a negative or strongly negative reaction. In all, 3.3% of participants expressed a neutral opinion regarding the issue of clarity in relation to the FoMO-R. The question regarding clarity is in Appendix A.

A variety of positive comments were received in relation to the clarity of the FoMO-R. The following is an outline of some of these:

“FoMO-R is straight forward”.

“I can describe FoMO-R as a self-explanatory method that enables a person to use it without professional help”.

“The guides were easy to read and follow”.

“The method was clearly explained and made easy to use”.

“It was explained very clearly and sufficiently”.

“The whole means of dealing with FoMO, alongside the stickers helped to countermeasure against FoMO”.

“The colours really helped”.

However, there was a negative comment about the background colour of materials. Some participants mentioned that “in some contexts, the background colour could be confusing, and they suggested using light colours to make the text easier to read”. This highlights the importance of graphic design in FoMO-R and that it shall be enhanced, taking into account how different people perceive colours and symbols.

#### 4.2.4. FoMO-R Coherence

**Figure 5:**
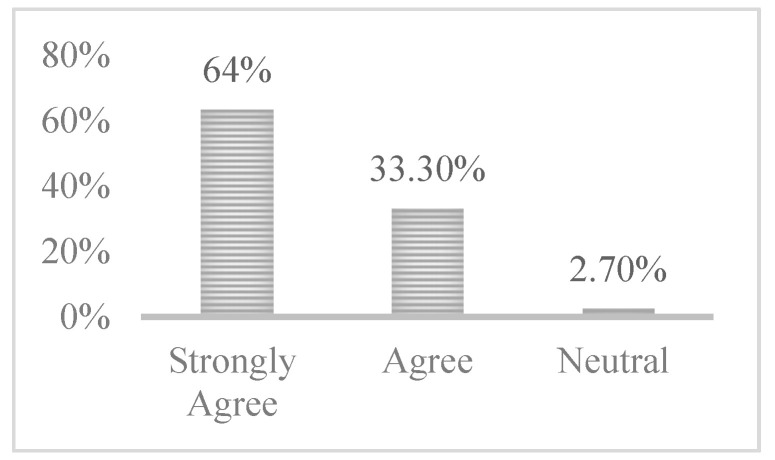
Coherence of FoMO-R.

FoMO-R coherence was another aspect examined in the evaluation study (see Appendix A). This component was included in order to establish whether each FoMO-R stage provides a solid foundation for the following one. From a participant perspective, questionnaire responses (see Figure 5) revealed that a similar proportion expressed either a strongly positive or a positive reaction, with 50% strongly agreeing and 46.7% agreeing. A further 3.3% of participants expressed a neutral opinion that strong continuity exists between all the FoMO-R stages. Positive comments were recorded regarding the coherence of the FoMO-R. The following are some examples:

“The structure was connected and when you follow the steps it makes sense”.

“They all link really well together”.

“They were all clearly linked and easy to follow”.

“From Booklet 1 to Booklet 4, it all flowed and made sense, easy to use and illustrative”.

#### 4.2.5. FoMO-R Usability

**Figure 6:**
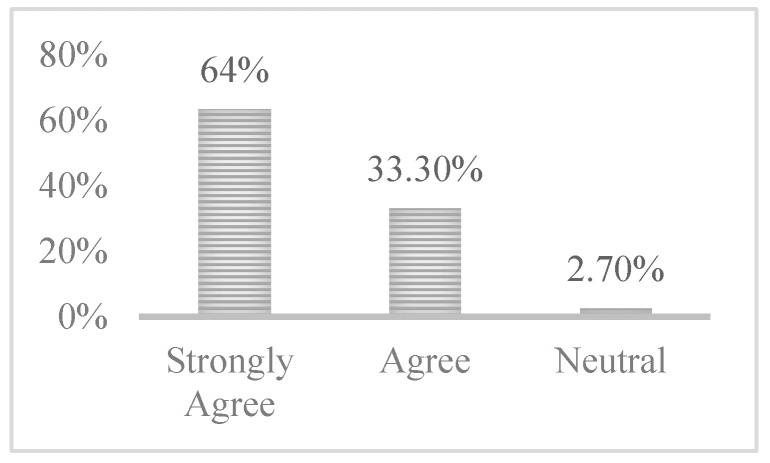
FoMO-R usability.

Usability in our evaluation focused on the ease of use and learnability of a tool. Within a software engineering context, usability is the level to which software can be utilised by specified individuals to perform particular goals with effectiveness, efficiency and satisfaction. Thus, the usability of the FoMO-R was evaluated and considered in this study (See Appendix A), as was the self-help guide which has also been developed to accompany it. In response to the question regarding FoMO-R usability, quantitative analysis (see Figure 6) revealed that most participants strongly agreed with the statement that the FoMO-R was not difficult to use (63.6%). In addition, it was noted that that a further 33.3% of participants expressed a positive reaction. A neutral opinion regarding FoMO-R usability was held by 3.3% of participants. A variety of positive comments were expressed regarding the usability of the FoMO-R. The following is a sample of some of them:

“Overall, the experience was efficient and effective. It was simple and clear and explained for easy implementation”.

“The method is very simple if the process was followed according to the steps provided”.

“It was a straightforward method to follow and presented clearly”.

“The stickers and different booklets made FoMO-R really easy to follow and use”.

#### 4.2.6. Effectiveness of FoMO-R

The effectiveness of FoMO-R was evaluated by undertaking a comparative analysis of the FoMO experience before and after using FoMO-R (see Appendix B). To achieve this, a series of paired sample t-tests were performed to compare the FoMO experiences of participants in different contexts both before and after FoMO-R usage.

In response to the question regarding the level of awareness of different FoMO types, a significant change was noted in relation to social media before using the FoMO-R (M = 3.43, SD = 1.00) as compared to after using it (M = 1.23, SD = 0.43); t (29) = 12.09, *p* < 0.001. In addition, in response to the question regarding the level of awareness of managing FoMO, a significant change in awareness levels occurred before using the FoMO-R (M = 3.56, SD = 1.16) as compared to after using it (M = 1.70, SD = 0.43); t (29) = 7.53, *p* < 0.001.

In Context (1), where others do not interact as expected, a significant change in FoMO experiences before using the FoMO-R (M = 21.13, SD = 8.05) and after its usage (M = 28.53, SD = 5.32) was recorded; t (29) = −6.35, *p* < 0.001. This was represented as an improvement in managing FoMO.

In Context (2), reflecting an inability to interact or connect as desired, a significant change in FoMO experiences before using the FoMO-R (M = 46.06, SD = 15.56) and after (M = 65.80, SD = 10.42) was recorded; t (29) = −10.13, *p* < 0.001. This was represented as an improvement in managing FoMO.

In Context (3), displaying an unwillingness to engage in social interaction, a significant change in FoMO experiences before using the FoMO-R (M = 19.03, SD = 7.86) and after using it (M = 28.53, SD = 5.41) was recorded; t (29) = −9.13, *p* < 0.001. This was represented as an improvement in managing FoMO.

In Context (4), referring to having to or feeling a need to engage in continuous and untimed interaction, a significant change in FoMO experiences before using the FoMO-R (M = 44.80, SD = 16.37) and after using it (M = 69.30, SD = 10.51) was recorded; t (29) = −10.45, *p* < 0.001. This was represented as an improvement in managing FoMO.

In Context (5), when an online social gathering is expected, a significant change in FoMO experiences before using the FoMO-R (M = 13.33, SD = 4.56) and after using it (M = 19.13, SD = 3.31) was recorded; t (29) = −8.26, *p* < 0.001. This was represented as an improvement in managing FoMO.

A Bonferroni correction was applied to the results to accommodate the multiple comparisons that were made to the data set (0.05/7). All significant results from the paired sample t-tests were <0.001, and as such the application of this correction did not change the interpretation of the results.

Overall, taken together, these findings suggest that the FoMO-R is expected to work effectively and contribute to helping people to manage their FoMO. This is achieved by creating awareness among them of how FoMO occurs on social media and how it can be managed by adopting different countermeasures, which can be either technical or social in nature.

#### 4.2.7. Engagement of Using FoMO-R

Engagement, within this context, refers to a willingness to use the FoMO-R, in the absence of any interpersonal coercion or pressure being exerted. This is a reflection of “autonomous behaviour which is one for which the regulation is experienced as chosen and as emanating from one’s self. In contrast, controlled behaviour is one for which the regulation is experienced as pressured or coerced by some interpersonal or intrapsychic force” [61]. Thus, TSRQ [60] was used to test the participants’ reasons for continuing to use the FoMO-R, as well as to adhere to the guidelines provided. The participants were required to complete this questionnaire after the third day of the study.

Table 7 presents the means and standard deviations of each factor for participants who completed the questionnaire. The first factor was autonomous regulation, and it comprised five items representing autonomous reasons for using the FoMO-R (Q4, Q6, Q7, Q9 and Q12) and adhering to the guidelines. The scores for this factor were in the range of 5 (low score) to 35 (high score). The second factor, controlled regulation, contained eight items, which described control-related reasons for using the FoMO-R (Q1, Q2, Q3, Q5, Q8, Q10, Q11 and Q13) and adhering to the guidelines. The score range for this factor was from 8 (low score) to 56 (high score). The descriptive statistics produced were as follows: autonomous regulation (M = 26.20, SD = 4.06); and controlled regulation (M = 30.43, SD = 9.73).

These results indicate that the mean autonomous regulation score was 26.20, which is a positive outcome, given that it is relatively close to the highest possible value of 35. This may reflect that participants were self-directed and purposefully engaged in using the FoMO-R, thus exercising a strong sense of choice. Essentially, participants continued to use this tool without feeling in any way pressurised or coerced. In contrast, their controlled regulation mean score was 30.43, which was much lower that the highest possible value of 56.

#### 4.2.8. Acceptance of FoMO-R

Acceptance of the use of the FoMO-R as a method to keep using in the future was tested using the e-TAP [62], which is based on the Theory of Planned Behaviour (TPB). Ajzen [63] devised the TPB, which is underpinned by a social cognition model and affirms the role of intention in anticipating actions [64]. This theory advances that intention in itself is the result of the following four factors:

*Behavioural intention, which refers to the motivational elements required to predict individual behaviour.*

*Attitude toward the behaviour, which is influenced by expectation and the desired outcomes.*

*Subjective norms (for example, social pressures and norms), which are determined by the expectations of peers and individual motivation to comply with their beliefs.*

*Perceived behavioural control, which refers to the perceived capacity to achieve the behaviour.*

Originally derived from the theory of reasoned action [65], this theory gradually evolved, whereby a third factor was inserted which expresses the same sense of self-efficacy [66]. Such factors can be used to create an exploratory framework which aims to predict people’s behaviours [67].

The e-TAP questionnaire was used to predict FoMO-R usage behaviour among participants. In order to achieve this, an e-TAP questionnaire was selected, which measures TAB factors. This questionnaire contained 16 items. Having used the FoMO-R, participants were then requested to complete the e-TAB questionnaire.

In response to the questionnaire, Table 8 outlines the distribution of means and standard deviations for intention, attitude, subjective norms and perceived control in relation to FoMO-R use. The score range for the behavioural intention subscale was between 4 and 28; however, the actual mean score recorded was 23.90 (SD = 2.74), indicating that the participants had moderate intention to use the FoMO-R. The participants displayed a favourable attitude toward use of the FoMO-R, as evident from their mean score of 25.36 (SD = 2.15). In relation to the subjective norms and perceived behavioural control subscales, the participants’ scores were 23.70 (SD = 2.53) and 23.93 (SD = 2.9), respectively. This indicates that the opinions of significant others regarding the participants’ FoMO-R usage behaviour, along with participants’ perceived control over FoMO-R usage, exerts a positive influence on the latter’s’ behaviour.

The results emerging from all e-TAP factors are positive, which indicates the possibility that the FoMO-R method will be adopted in the future. Essentially, participants perceive that the FoMO-R is a useful approach in helping them to manage their FoMO more effectively.

## 5. Discussion

In this paper, we proposed and evaluated FoMO-R, a method to help people to manage FoMO. The method was proposed based on previous findings on the types of FoMO, their context and countermeasures. We also added a new set of countermeasures related to skills and practices on social media. The theories and models explained in Section 2 provided theoretical underpinnings for the FoMO-R method. Resilience was studied as FoMO-R is designed to make people who use social media aware of how FoMO happens (preparation stage, using Booklet 1) and how FoMO is managed (planning stage, using Booklet 2) and, thus, building awareness and resilience in them. Self-control theory relates to FoMO-R, as the method helps people to control their FoMO by using technical or socio-technical countermeasures, mainly at the planning stage, using Booklet 2. Self-talk, self-esteem, expectations management, uncertainty avoidance, anxiety management and compulsive behaviour can be seen in the planning stage when using Booklet 2, employing social and personal countermeasures. Self-talk is used also in the empowerment stage, in Booklet 4. The transtheoretical model was taken as a template for the FoMO-R stages.

We evaluated FoMO-R through a three-step process. The first step involved collecting baseline information; the second step entailed applying FoMO-R tools and advised practices for ten days, and the third step was to complete the paper survey. The sample size was 30 participants. The participants were asked to apply FoMO-R and provide any suggestions they may have. The analysis was conducted using descriptive analysis (frequency procedure) for each statement independently regarding aspects such as clarity, coherence, coverage and usability. The results show that the majority of participants had a positive attitude towards these aspects after using FoMO-R. Paired sample t-tests were conducted on the total score within each context of felt FoMO, before and after using FoMO-R. As such, the quantitative data that were collected and analysed appeared to triangulate and support the qualitative results, although as discussed in more detail below it is acknowledged that the sample size used in the study limits how strongly this claim can be made. The results show that FoMO-R was anticipated to help people to effectively manage their FoMO. This is achieved by creating awareness among them about how FoMO happens on social media and how they can be managed by adopting different countermeasures which can be either technical or social in nature.

Two of the participants commented on the amount of information in the booklet. One said “*there is a high volume of information provided by FoMO-R materials, and this may make us a little bit confused*.” However, a balance between the wealth of information and the cognitive load it might lead to is hard to achieve. In our future work, we will look for personalization in the material itself to reduce what can be seen as trivial or redundant. FoMO-R needs essential information, especially about the types of FoMO experienced, and reducing this diagnosis part can be challenging. In the future, this issue can be reduced when FoMO-R is implemented as an online app, allowing navigation and filtering. This means that the amount of information can be decreased and customised based on a user’s FoMO type(s).

While the participants indicated that FoMO-R was easy to use, it was observed that the first impression of the participants when they saw the FoMO-R material was that it was overwhelming. However, the participants did not comment on this further after they started to use the method. It indeed acted as an educational source about FoMO and countermeasures in general and that seems to have helped literacy around the topic.

Coherence was one of the aspects that was considered in the FoMO-R evaluation. Two of the participants had a negative attitude regarding the level of coherence; they argued that some of the loops inside certain stages could be confusing, and they suggested that the number of loops should be decreased. This would make it easier for them to achieve the stages smoothly. Reducing the number of loops could adversely affect the effectiveness of FoMO-R. The control loop depends on the outcome to refine the input, and this also applies when people try to change behaviour. However, by using AI and personalization, we may still be able to get the right countermeasures and skills for relapse prevention through a limited number of loops.

Another potential approach is to engage representative help-seekers in co-design sessions with researchers to tailor the proposed countermeasures the social network nature and features and its typical scenarios and contexts of use. For example, FoMO on instant messaging has a different nature and patterns on online forums. Using such a democratic technique can bring in-depth insight into the method design, and thus allowing a better understanding of the target population and a more effective design of the countermeasures [68,69].

A challenging aspect that three of the participants referred to while using FoMO-R is the pressure that they felt from peers and significant others (subjective norms). For example, when they set their status to say that they are unavailable, the messages from their peers or family continued. This could make them feel under pressure to respond in order to maintain relations and not suffer a loss of relatedness or popularity. However, some of the participants tended to use the self-talk technique to help alleviate such pressure; e.g., *“I set my status saying I am busy at the moment, so I do not need to check each notification immediately”.* This highlights the challenge when users try to regulate their relationship with social media as it is often linked to others’ reactions and their groups’ norms. Applying some of our countermeasures can, in turn, trigger other FoMO of the type, “what would others say about me as I changed my status to unavailable”.

Although our main purpose in this paper is to devise and introduce FoMO-R and that our evaluation study is mainly meant to provide evidence of the potential of FoMO-R, we still recognize that this evaluation has limitations:

The time of applying FoMO-R was short for judging whether it has led to a sustainable change in FoMO feeling and management in the participants. However, the authors wanted to establish evidence of whether FoMO-R was accepted in the first place and that there is an intention to use. Ensuring long term adoption and sustainable change will need a longer time period.

The sample size for this study was 30 participants, and this would be considered a small sample size. A larger sample size would help to get better insights and more comments to enhance the FoMO-R. In future studies, the implementation of FoMO-R will involve an online application, e.g., through delivering the method via an online app that is handy to access and that also allows the activation of certain countermeasures directly, e.g., the mute of notification and the automatic set of status and auto-replies. The study relied upon self-report for both the quantitative and qualitative data collection. We acknowledge the issues that can arise from self-report, although we would note that it has been observed with reference to other compulsive behaviours that self-report can be more reliable than may typically be envisaged [70]. In addition, our study was demanding in terms of time, lasting for ten days, and in terms of effort, requiring the filling in a diary template. We would expect that the voluntary continuation in the study is another reason to believe in the truthful nature of the participants’ answers.

Some of the technical countermeasures, such as recap and the priority list, have not yet been implemented fully on de facto social media. If participants want to use them during our study, this could affect the full utilisation of FoMO-R. However, we tried to overcome this limitation by providing participants with alternatives which may work similarly to these technical countermeasures; for example, muting the notifications from unwanted contacts or groups as a way to personalise and filter notifications.

Since the participants were given a reward in return for their participation in our evaluation study, this may have influenced their feedback or answers and affected the trustworthiness of the data. However, rewards are commonly utilised in research and participants were asked to provide their own opinions even when those opinions were negative. In addition, there were no further interviews or face-to-face sessions after they were given the templates and asked to fill in and return. The reward was given upon the return of the forms. It is, therefore, unlikely that the giving of rewards actually affected the participants’ answers. We also believe social desirability bias has been minimised as much as possible through reiterating to the participants that we are looking for their true feelings and thoughts about the method and that there are no wrong or right answers.

## 6. Conclusions

In this paper, we proposed a method, FoMO-R, for helping people in managing FoMO. The method is based on previous research that the authors did and in which they discovered the lived experience of FoMO, the contexts of use leading to it and the fears encountered. Our previous research also identified technical countermeasures for FoMO. In this paper, we introduced further skills and practices that help the users in managing FoMO and mainly through managing other contacts’ expectations and their pressure. FoMO-R was evaluated in terms of its effectiveness, clarity, coherence, usability, engagement and acceptance. The results of the evaluation study indicate positive attitudes towards the ability of FoMO-R to help participants to manage their various kinds of FoMO. In our future work, FoMO-R will be assisted via software so that it integrates well with computing devices and social media. For example, we plan to equip it with the ability to monitor usage, with user consent, and contrast that with reported FoMO types and usage styles. We also plan to gamify the method, so that information and tests are seen as less demanding in terms of time. Some of the countermeasures we propose are reliant one whether social media platforms and operating systems allow for them to be implemented and integrated. We plan to provide evidence that they can yield a healthier relationship with technology as a way to motivate their introduction in the future.

## Figures and Tables

**Table 1:** FoMO: context classifications, fears and countermeasures (summary of [2,18,19]).

Classification 1: FoMO When Others Do Not Interact as Expected		
Kinds of FoMO	Preoccupied with	Countermeasures
Fear of missing the ability to be popular	Lack of participation Missing prior interactions Loss of reputation	Autoreply Set status
Fear of missing the ability to be interesting	The post is not appealing enough Profile less active	Set status Auto view
Fear of missing the ability to get the right interpretation	Whether the message is delivered Whether the message has been understood	Autoreply Set status
**Classification 2: FoMO When Unable to Interact or Connect as Wished**		
Fear of missing information due to large volume	Missing a particular post Reaching the necessary information	Filter Priority list
Fear of missing the ability to deal with different social networks	Locating an important message Prioritising the response	Auto-status Set status
Fear of missing temporally available information	Removed by a person or naturally disappeared Frustration with speed versus temporal availability information Frustration with limited data usage versus temporal availability information	Event and content record Diverse notification
Fear of missing a timely interaction	Immediate response-Misunderstanding (friends feel they are ignored) Give negative impressions	Autoreply Set status
Fear of missing the ability to keep followers	Increase participation on SNSs Need to update the profile frequently	Set status Contextual Awareness
Fear of missing information/events due to multi following	Missing post from a certain person (celebrities)	Priority list Filter
**Classification 3: FoMO When Unwilling to Engage in Social Interaction**		
Fear of missing valuable information	Ad hoc requests Lose the benefits of the group	Terms and conditions Recap
Fear of missing the ability to defend your popularity	Misunderstanding (i.e., ignoring friends)	Self-expression Set status
**Classification 4: FoMO When Having to or Feeling a Need to Engage in Continuous Untimed Interactions**		
Fear of missing empathy and leaving a good impression	Not interested in the conversation Missing self-image (rude) 8Missing empathy Making others think there is something wrong Hurting others’ feelings (e.g., affect others’ self-esteem)	Self-expression Set status
Fear of missing the opportunity to know others’ impressions	Need to reply Need for appreciation Need to delete the post if negative comments are given	Priority list Diverse notifications
Fear of losing popularity	Reply immediately Not letting people feel ignored	Set status Alternative notification
Fear of missing a valuable opportunity	Missing commercials Missing employment opportunities	Diverse notifications Alternative notification
Fear of missing the sense of relatedness	Ad hoc requests Missing what is going on in others’ lives	Colour indication Contextual Awareness
Fear of missing spontaneous responses	More information needed from the sender	Diverse notification Alternative notification
**Classification 5: FoMO When an Online Social Gathering is Expected**		
Fear of missing the opportunity to attend an online event	Missing the live chat	Calendar event reminder Recap
Fear of missing the sense of relatedness	Missing peoples’ availability on social media	Terms and conditions Colour indication
Fear of missing the ability to be popular	Missing social rank	Set status Terms and conditions

**Table 2:** FoMO-R stages (booklets are in the Supplementary Material of this paper).

No	Stage	Description	Strategies to Guide Change
1	Preparation	This stage raises awareness about FoMO and also provides the help-seeker with a greater understanding of the types of FoMO.	Help-seeker selects the FoMO type(s) that they typically experience by referring to the self-rating booklet (Booklet 1). They remove the sticker(s) for their selection(s) and post it(them) on the self-monitoring sheet.
2	Planning	This stage assists the help-seeker to plan to manage FoMO by providing them with a set of suggested countermeasures. This includes learning:Technical countermeasures, e.g., auto reply, set status, etc.Socio countermeasures, e.g., managing expectations, self-talk, self-control, etc.Relapse prevention strategies which will prevent the help-seeker from reverting to their old habits.	For each type of FoMO that was selected on the self-rating booklet (Booklet 1), help-seeker selects suitable technical and/or socio countermeasure(s) from the list of FoMO-reduction countermeasures document (Booklet 2). Help-seeker removes the sticker(s) for their selection(s) and posts it (them) on the self-monitoring sheet. Help-seeker learns about relapse using Booklet 3.
3	Action	Help-seeker applies the selected countermeasure.	Help-seeker applies each of their selected countermeasures for a period of time, typically one week. To prevent relapse whilst applying their selected countermeasure(s) they need to perform the skills or activities outlined in Booklet 3; e.g., hobbies, positive self-talk, seeking moral support.
4	Assessment	Help-seeker assesses their satisfaction with the selected countermeasure(s).	For each countermeasure they selected for each of the FoMO types they have, the help-seeker indicates on the self-monitoring sheet whether it was useful for them. If they found at least one useful countermeasure for each of their FoMO types, they go to Stage 4; the review stage. If none of the countermeasures for one or more of the FoMO types they have failed to work, they go to the empowerment step.
5	Empowerment	It provides those seeking help with further support to manage their FoMO in the event that the countermeasures selected from Booklet 2 fail	Help-seeker determines the challenges that may make it harder for them to manage their FoMO by answering the following questions: Is peer pressure the cause? Do you put the needs of others above your own? Are there technical issues? They select other countermeasures from the FoMO countermeasure document (Booklet 2) or follow the instructions in the empowered refusal document Booklet 4. Return to the application stage and repeat. If they run out of countermeasures and are unable to cope with their FoMO, go to the review stage.
6	Review	Help-seeker reviews their self-monitoring sheet to assess whether they have managed their FoMO.	Help-seeker describes the outcome of actions taken by answering the following: What happened? What has been improved? Did you manage your FoMO? If help-seeker managed their FoMO, they repeat the first stage to see whether they have any other types of FoMO that could be addressed. If not, they stop. If they failed to manage their FoMO, they check that they had: Selected the types of FoMO that applied to them. Maintained a focus on the selected countermeasures and on the appropriate relapse prevention technique(s). If, after this, they have seen no improvement, there may be comorbidities.

**Table 3:** Self-rating (Booklet 1 in the Supplementary Material).

When Others Do Not Interact with Me as Expected on Social Media, I May Be:
Preoccupied with the lack of my participation with others that leads them not to interact with me (P1)
Preoccupied with missing prior interactions with others that leads them not to interact with me (P2)
Preoccupied with the loss of my reputation among friends (P3)
Preoccupied with my post not being appealing enough (P4)
Preoccupied with my profile being less active so that others do not interact with me (P5)
Preoccupied that living outside my geographic social circle leads them not to interact with me (P6)

**Table 4:** Sample of FoMO countermeasures for Context 1 (Booklet 2 in the Supplementary Material).

Context 1: FoMO When Others Do Not Interact as Expected		
You posted your holiday pictures on social media a few times and expected to receive some ‘Likes’ and comments from friends, but you did not receive any. Thus, you may experience the following FoMO but may practice the following techniques to reduce your fear:		
**Kinds of Fear**	**Recommended Technical Countermeasures**	**Recommended Socio-Technical Countermeasures**
Fear of missing the ability to be popular (P1, P2 and P3)	Auto-reply; e.g., when you send a message, you would like your contacts to set a form of autoreply to inform if they cannot interact currently Set status; e.g., before you send the message, you would be happy if your contacts declare their status; e.g., busy, driving, on a call, etc. so you know they may not respond swiftly	Try to practice self-talk, you can say: *I do not expect interaction from others when I post on social media* Try to improve your self-esteem by saying*: I am not the only one who does not receive the interaction they expect* Try to manage your expectations by *posting on social media without expecting interaction from others OR interact with others without expecting reciprocal interactions* Try to control your anxiety by *distracting yourself by engaging in offline activities such as making coffee, cleaning the house, talking to the person next to you, walking around your house, etc.* Try to ask yourself what you would say to a friend who faced a similar situation
Fear of missing the ability to be interesting (P4, P5 and P6)	Set status: you would like certain contacts to set their status in advance to show you whether they are online or available to interact or not. Auto views: social media shows you who viewed your post	

**Table 5:** Sample of relapse prevention (Booklet 3 in the Supplementary Material).

What is relapse? Relapse “refers to a breakdown or failure in a person’s attempt to change or modify any target behaviour.” What is relapse prevention? Relapse prevention (RP) is a self-control technique designed to help individuals who are trying to change their behaviour. It enables them to anticipate and cope with the problem of relapse. Important points, please read them: If you manage your FoMO for a while and then begin to experience it again, you have relapsed. Relapse happens because people may stop using the FoMO reduction method. Relapse can be avoided by will power and self-discipline alone. Relapse prevention plan Coping skills: please list activities or skills you enjoy that will take your mind off social media 1. 2. 3. 4. 5. To prevent relapse: Please do one or more of the activities or skills that you listed in the relapse prevention plan

**Table 6:** FoMO-R evaluation process.

Step	Activity
Before the use of FoMO-R	Measuring FoMO awareness and experience before using FoMO-R.
During the use of FoMO-R	Completing the diary template. Measuring the engagement in FoMO-R using a treatment questionnaire concerning continued programme participation (TSRQ).
After the use of FoMO-R	Measuring FoMO experiences after using the FoMO-R. Measuring the intention to use the FoMO-R using e-Therapy Attitudes and Process Questionnaire (e-TAP). Questioning the participants for their perceived FoMO-R usefulness, coverage, coherence, clarity and the coverage of FoMO-R materials.

**Table 7:** Means, standard deviations, ranges, and sample sizes for autonomous regulation and controlled regulation.

Factors	*N*	No. Item	Possible Scores	Mean	Std. Deviation
Autonomous Regulation	30	5	5–35	26.20	4.06
Controlled Regulation	30	8	8–56	30.43	9.73

**Table 8:** Distribution of range, means and standard deviation of intention, attitude, subjective norm, and perceived control as predictors of FoMO-R use (*n* = 30).

Factors	N	No. Item	Possible Scores	Variance	Mean	Std. Deviation
Behavioural Intention	30	4	4–28	7.54	23.90	2.74
Attitude toward the Behaviour	30	4	4–28	4.65	25.36	2.15
Subjective Norm	30	4	4–28	6.42	23.70	2.53
Perceived behavioural Control	30	4	4–28	8.47	23.93	2.911

## Supplementary Materials

The following are available online at https://www.mdpi.com/1660-4601/17/17/6128/s1, Booklet 1: Self-rating booklet, Booklet 2: List of FoMO countermeasures, Booklet 3: Relapse prevention, Booklet 4: Empowered refusal and Self-talk.

## Appendix A. Assessment of FoMO-R and Its Materials

A 5-point Likert scale reflecting an agreement was used for this survey with open-ended questions for each question to enable the participants to elaborate their answer. The questions are as follows:

Please answer the following questions about documents used in FoMO-R: I got sufficient information about FoMO that is provided by a self-help guide booklet (Coverage); I got sufficient information about how different FoMOs happen on social media that is provided by self-rating booklet (Booklet 1 Coverage); I got sufficient information about the countermeasures for combating FoMO that is provided by FoMO-reduction countermeasures booklet (Booklet 2 Coverage); I got sufficient information about relapse prevention booklet (Booklet 3 Coverage); I got sufficient information about empowerment further support that is provided by Empowerment booklet (Booklet 4 Coverage);

Please answer the following questions about FoMO-R as a whole: I got sufficient information regarding how to use FOMO-R method; Generally speaking, FOMO-R method was not difficult to understand (e.g., it was explained in a clear way) (FoMO-R Clarity); Each stage of FoMO-R provides a solid foundation for the following one (FoMO-R Coherence); Overall, FOMO-R method was not difficult to use (FoMO-R Usability).

Optional question: Would you like to add any further thoughts? If yes, please explain?

## Appendix B. Survey Questions Relevant to FoMO Experience before and after Using FoMO-R

Demographics, the awareness level of knowing and managing FoMO types, and FoMO experience

What is your age?

What is your gender? Male; Female; Prefer not to say.

How do you rate your awareness of knowing the types of FoMO? Extremely aware; Moderately aware; Somewhat aware; Slightly aware; Not at all aware.

How do you rate your awareness of managing your FoMO? Extremely aware; Moderately aware; Somewhat aware; Slightly aware; Not at all aware.

45 FoMO experience questions Using the 1 to 6 scale, starting from Always to Never. Please tick the box as close as possible to your daily experience with FoMO on social media. The questions are as follows, and they are based on [2]

**Context 1: When others do not interact with me as expected on social media, I may be:**

- Concerned that others are choosing not to reply to me or comment on my post
- Concerned with missing to reply to others previous message or comment on their previous post that cause them not to interact with me
- Concerned that my reputation among my friends has declined
- Concerned that my post was not appealing enough for them to want to Like or Retweet it or even comment on it
- Concerned with my profile being less active so that others lose interest
- Concerned that if people live outside my city or country, they may not interact with me

**Context 2: When I am unable to interact or connect to social media as you wish, i may be:**

- Concerned with missing a certain post because of the large number of posts or tweets
- Concerned with the difficulty of reaching the posts or tweets I need because of the large number of posts or tweets
- Concerned with locating an important message because I have messages from many different social media users
- Concerned with responding the most important messages received from different social media users
- Concerned that other people’s posts may be removed or disappear; e.g., stories on Snapchat, urgent messages or news feeds that disappear after a period of time
- Concerned that I have to respond immediately to messages
- Concerned that my friends feel they are ignored
- Concerned with missing people who do not often post messages on social media so that I may not find them when I come back online
- Concerned with missing being involved in current discussions that may be interpreted to mean that I do not like to participate in friends’ discussions
- Concerned I may miss supporting or defending friends, teams or opinions on Facebook, WhatsApp, Snapchat, Twitter, Instagram or others social media platforms
- Concerned at the inability to talk regarding missing online discussions when my friends are gathered at school, restaurants, coffee shops, home, etc.
- Concerned with the need to increase my activity on social media to keep or increase my followers
- Concerned with the need to update my profile frequently to keep or increase my followers
- Concerned at missing posts from a particular person (e.g., celebrities)

**Context 3: When I am unwilling to engage in social interaction (e.g., group chat), i may be:**

- Concerned about missing unexpected requests from group members
- Concerned about losing the benefits of being in the online group (e.g., plans for a party, assignment information)
- Concerned that group members will not respond to me in future
- Concerned that my friends feel they have been ignored
- Concerned about damaging relationships and reputations with others
- Concerned that I will not be involved in future discussions in online groups

**Context 4: When I keep checking or feel a need to engage in continuous untimed interactions, I may be:**

- Concerned about reassuring friends I am interested in their conversation when I really want to stop the conversation
- Concerned about not harming my self-image when I really want to stop the conversation
- Concerned about not showing empathy when I really want to stop the conversation
- Concerned about not hurting other people’s feelings (e.g., affect others’ self-esteem) when I really want to stop the conversation
- Concerned about missing my social relationships
- Concerned about the need to reply to people who comment on my posts or tweets
- Concerned about the need to value people who comment on my posts or tweets
- Concerned about the need to delete a post if negative comments are made
- Concerned about doing something wrong before knowing others’ impressions (making wrong decisions) when I asked them about something

**Context 5: When I expect an online social gathering, I may be:**

- Concerned about missing live chats
- Concerned about being unaware of whether or not people are available on social media
- Concerned about whether or not my name is mentioned in online groups
- Concerned about losing influence among my friends
