# Supplementary material for: Combating Fear of Missing Out (FoMO) on Social Media: The FoMO-R Method
Source: Int J Environ Res Public Health. 2020 Aug 23;17(17):6128. doi: 10.3390/ijerph17176128 (PMC7504117; doi:10.3390/ijerph17176128)
Supplement: Supplementary file 1 [file ijerph-17-06128-s001.pdf]

Tackling the Fear of Missing Out (FoMO) when using social media:

A SELF-HELP GUIDE

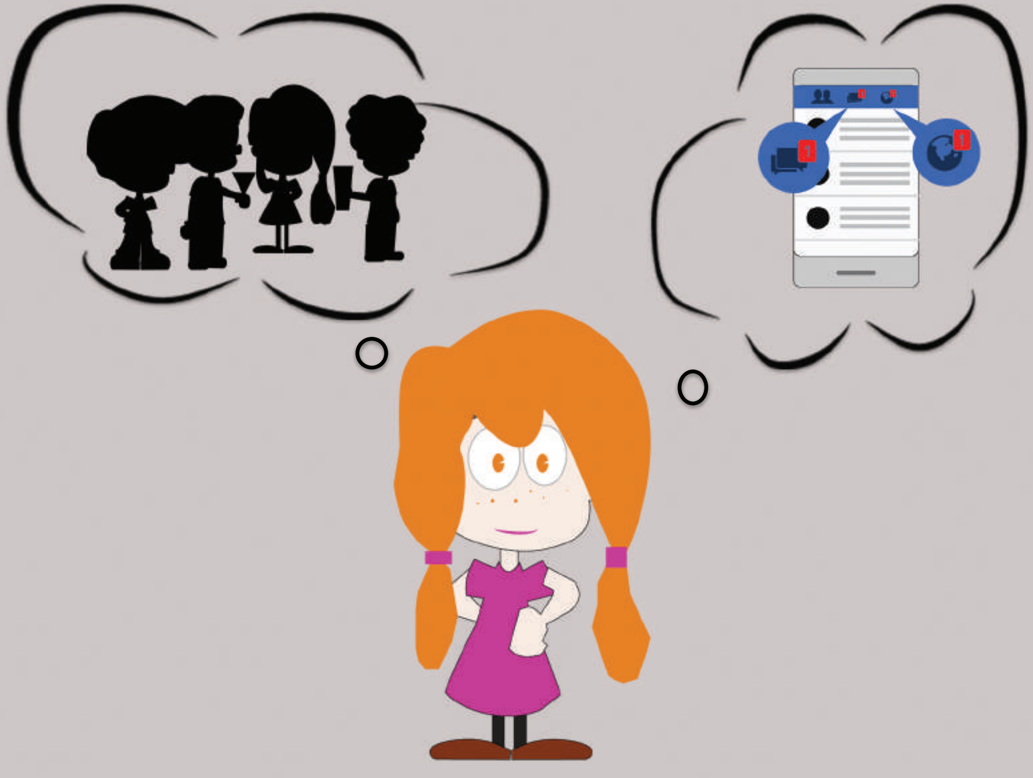

WHAT IS THE FOMO?

FoMO is something we all experience from time to time, including times when we use social media such as Facebook, WhatsApp, Twitter, Snapchat, Instagram, YouTube, WeChat etc. It's a feeling or a state of mind in which someone feels negative emotion such as anxiety when they believe they are missing out on something. It can often be linked to how much we connect to other people and certain content on social media. FoMO typically refers to a need to connect more with other people who are on social media platforms, and avoid losing those connections by being active online most of the time, posting content regularly, and communicating with other users often.

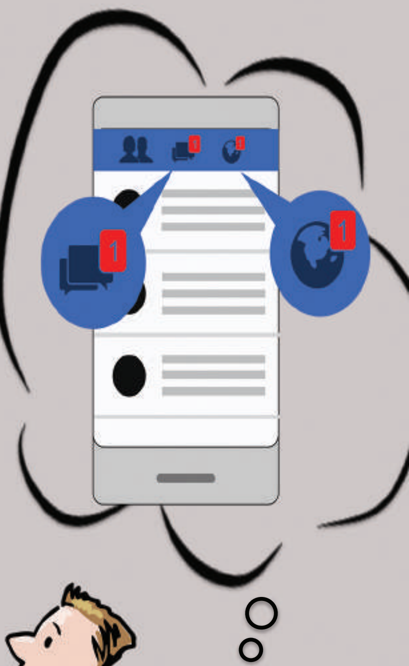

EXEMPLAR CASE

You are in a formal meeting and are unable to check your social media, even when you receive notifications. And even though you had a 5 minute coffee break it wasn't enough time to check all of your notifications and respond to everyone. Therefore, you may be concerned that you have to respond immediately to messages.

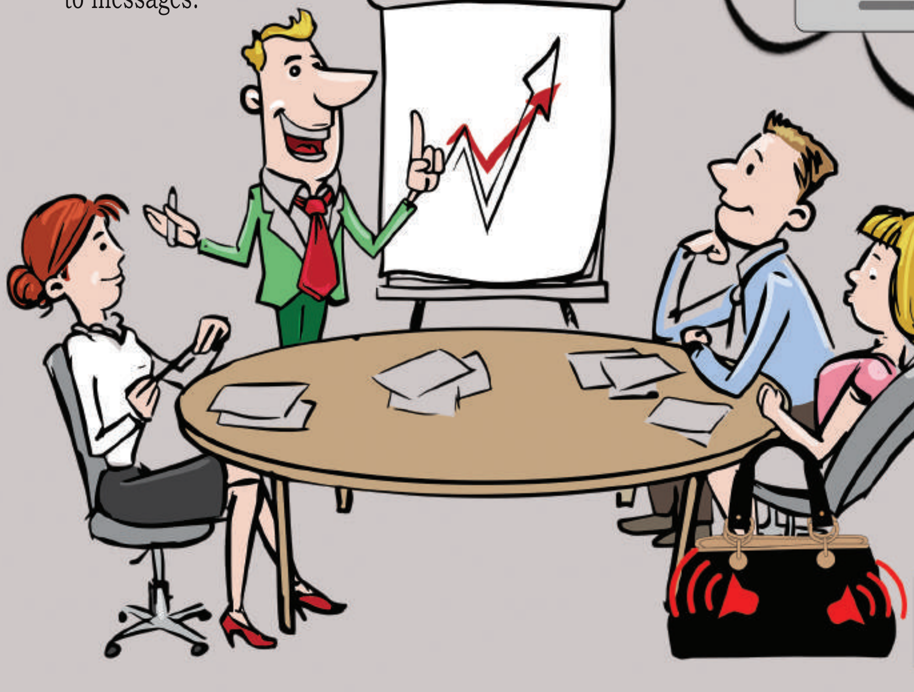

AM I EXPERIENCING FOMO?

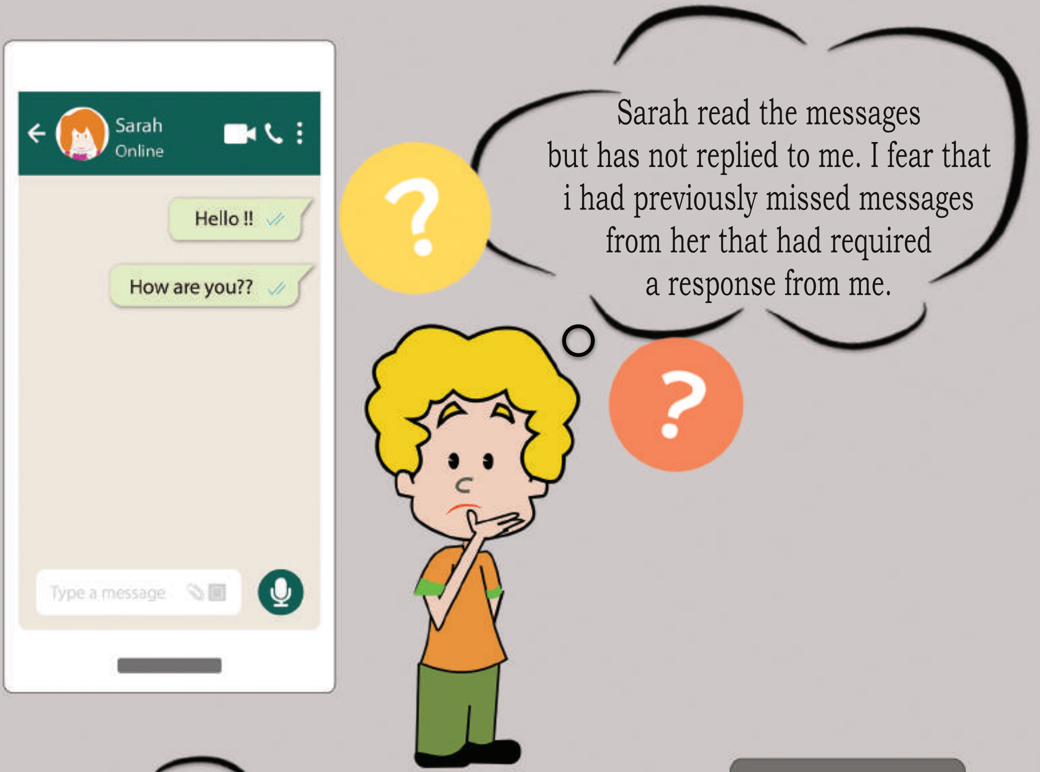

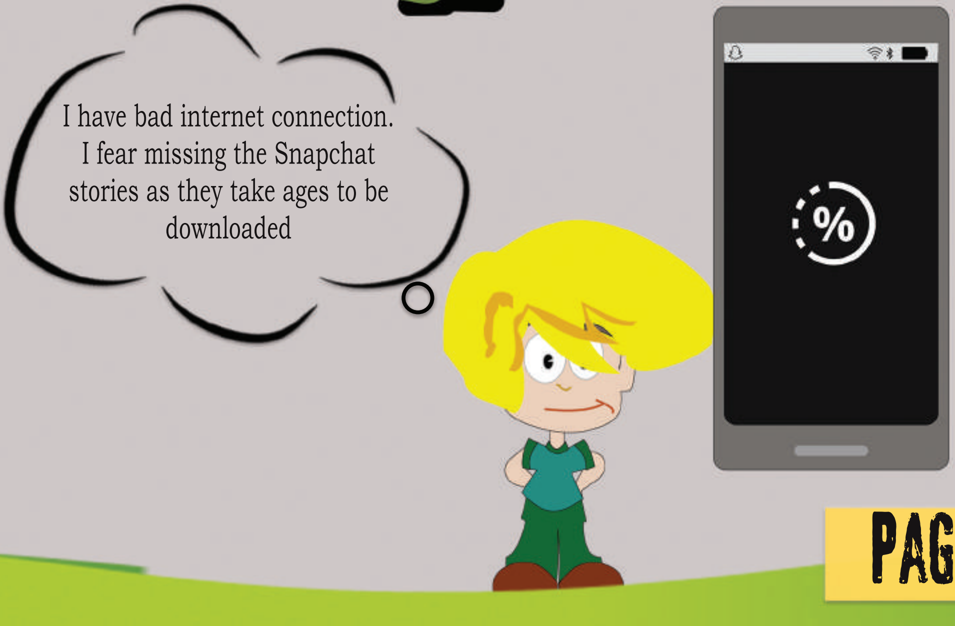

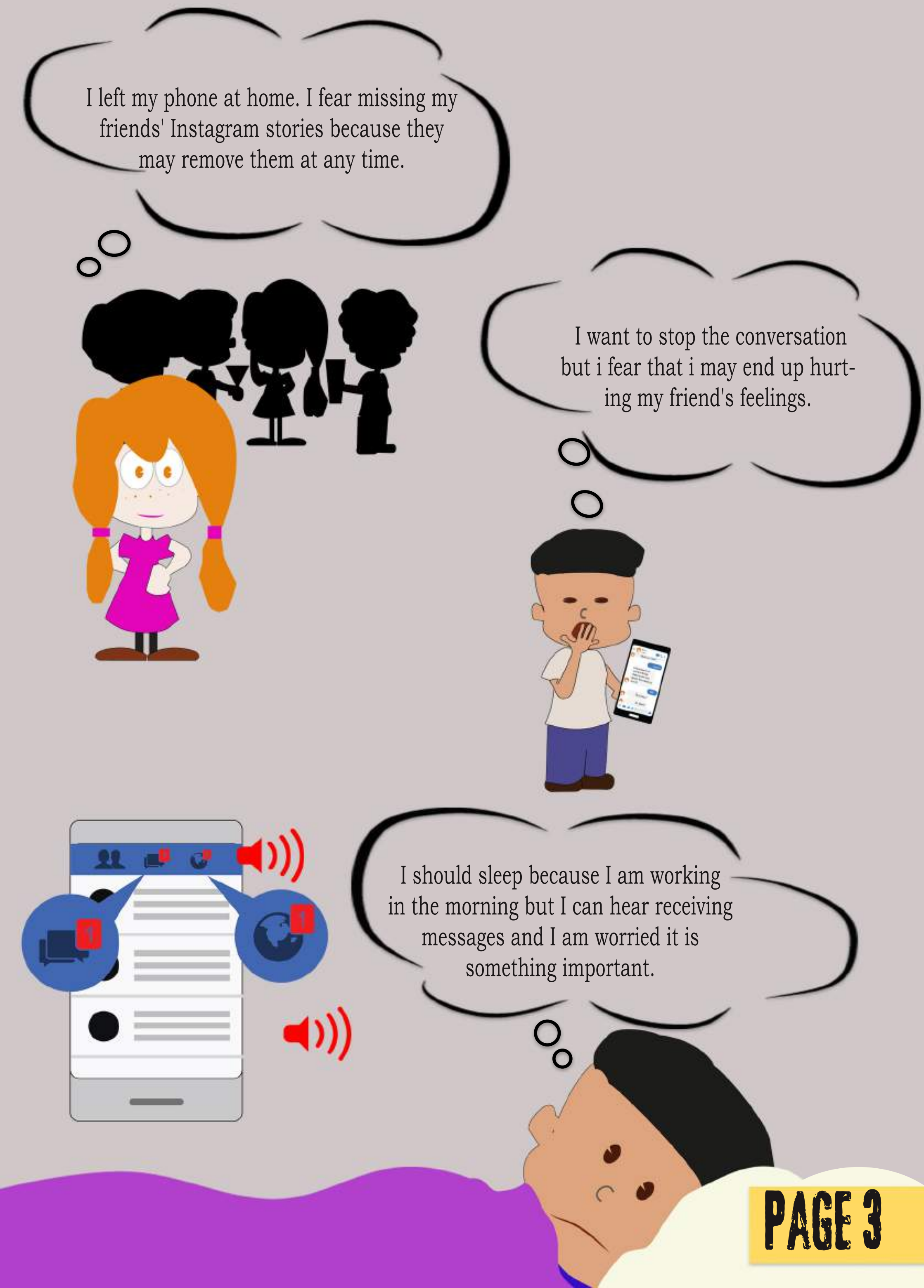
I left my phone at home. I fear missing my friends' Instagram stories because they may remove them at any time.
I want to stop the conversation but i fear that i may end up hurting my friend's feelings.
I should sleep because I am working in the morning but I can hear receiving messages and I am worried it is something important.

The previous examples are some common experiences which are faced by people who suffer from FoMO. If you experience FoMO you may have similar thoughts yourself. Sometimes it is possible to be experiencing FoMO and not even know it. Therefore, the first step in helping you to manage your FoMO is by recognising what type of FoMO you are experiencing. Thus, we have proposed the FoMO reduction method to help you to become more aware of and resilient to FoMO when social media triggers you to feel it.

FOMO REDUCTION METHOD (FOMO-R)

FoMO-R is a method that aims to reduce how much FoMO affects you. It helps you to recognise which types of FoMO apply to you when you use social media. By finding out your types you can then find the best techniques to help you manage FoMO. It involves a number of stages, and the following section explains the stages of FoMO-R that you need to follow in order to help you control how FoMO affects you.

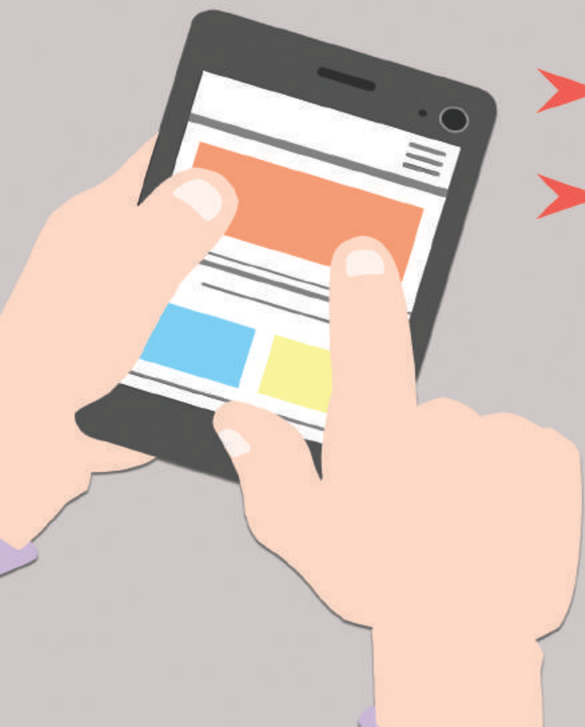

HOW TO USE (FOMO-R):

- Follow the stages of the reduction method.
- Fill in the FoMO Monitoring Sheet.

STAGES OF THE FOMO REDUCTION METHOD (FOMO-R)

In the process of managing FoMO on social media, the person who is seeking help may follow the stages in order to find out their types of FoMO, and then they may select the most suitable countermeasures that will help them to tackle their FoMO.

Note: Please use the FoMO Monitoring Sheet to post your selections (i.e. stickers).

1 PREPARATION

This stage is meant to raise awareness of FoMO, and also provides you with greater understanding about the various types of FoMO.

2 PLANNING

This stage is meant to help you plan toward your goal of managing FoMO by providing you with a set of suggested countermeasures. This includes learning:

- Technical countermeasures such as auto reply, set status, etc.
- Socio countermeasures such as self-talk, managing other's expectations, self-control, etc.
- Relapse prevention strategy, which will help you avoid going back to old habits instead of making progress.

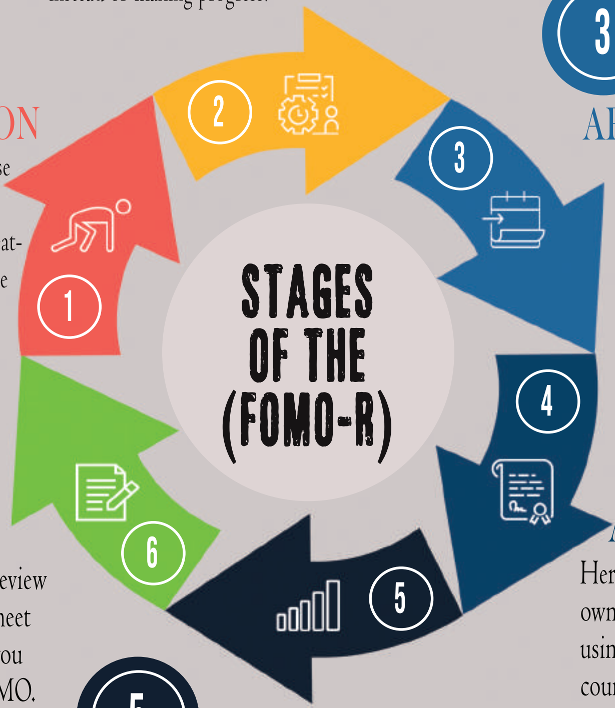

3 APPLICATION

Here you can apply the selected countermeasure

4 ASSESSMENT

Here you can assess your own satisfaction from using the selected countermeasure(s).

5 EMPOWERMENT

Empowerment of further support

6 REVIEW

This stage makes you review your self-monitoring sheet to see whether or not you have managed your FoMO.

STRATEGIES FOR EACH STAGE

1 PREPARATION

- Please select the FoMO type(s) that you typically experience by referring to self-rating sheet (Booklet 1).
- Please remove the sticker of your selection(s) and post it on self-monitoring sheet, and then go to the next stage; the planning stage.

2 PLANNING

- Please select the suitable technical or/and socio countermeasure(s) from the list of FoMO countermeasures document (Booklet 2) for each type of FoMO that was selected on the self-rating sheet (Booklet1).
- Please remove the sticker of your selection(s) and post it on self-monitoring sheet.
- Find out information about relapse using Booklet 3, and then go to the next stage; action stage

3 APPLICATION

- Please practice each of your selected countermeasures for a period of time typically one week.
- In order to prevent relapse from happening while applying your selected countermeasure you need to:

\- Perform the skills or activities that you were provided with on Booklet 3; e.g. doing hobbies, talking to yourself positively, seeking moral support.

- Please go to the next stage; assessment stage

4 ASSESSMENT

For each countermeasure you selected for each of the FoMO you have, please indicate whether it was useful for you using self-monitoring sheet.

\- If you found at least one useful countermeasure for each of your FoMO types, go to stage 6, the review stage.

\- If you have one or more of the FoMO types without any useful countermeasures, go to the next Empowerment stage

5 EMPOWERMENT

- Please determine the challenges that may make it harder for you to manage your FoMO by answering the following questions:

Is it peer pressure? | Is it due to you putting others needs above your own? | Is it technical issues?

- Select other countermeasures from the FoMO countermeasure document (Booklet 2) and Please remove the sticker of your selection(s) and post it on self-monitoring sheet, OR—
- Follow the instructions in Booklet 4 and post your selection on the self-monitoring sheet.
- Return to the application stage and repeat.
- If you run out of the countermeasures and unable to cope with your FoMO, go to the next stage; review stage

6 REVIEW

- Describe the outcome of your actions by answering the following on self-monitoring sheet:

What happened? | What has been improved? | Did you manage your FoMO?

- If you managed your FoMO, please repeat the first stage; preparation stage to see if there is any other FoMO types that applies to you. If instead you do not select any types of FoMO again you can stop.

If you did not manage your FoMO, please ensure:

1. You selected FoMO types that apply to you.
2. You kept your focus on applying you selected countermeasures alongside with relapse prevention technique

- If you have not seen any improve, this can be that you may have comorbidity.

BOOKLET 1

Self-rating booklet

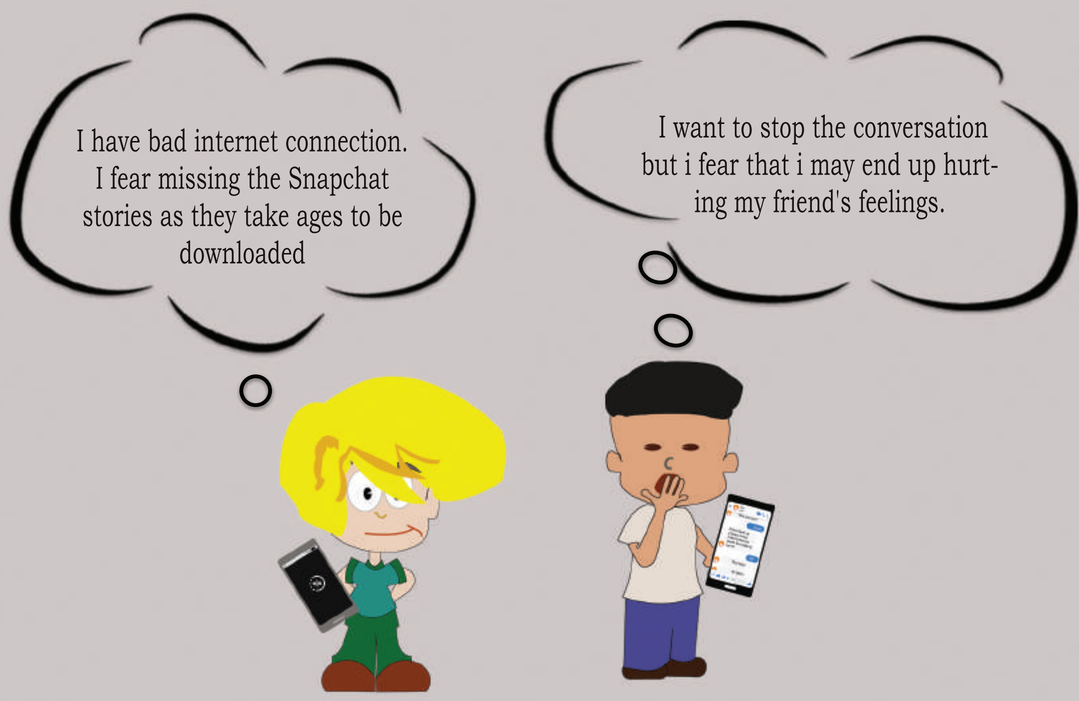

Below is a set of statements which may describe your everyday experience with social media. Please select the statement(s)that really reflects your typical experiences and then remove the sticker from the sticker sheet based on your selection. Please answer according to what really reflects your experiences rather than what you think your experiences should be:

Context1: When others do not interact with me as expected on social media, I may be:

P1
Concerned that others are choosing not to reply to me or comment on my post

P2
Concerned with missing to reply to others previous message or comment on their previous post that cause them not to interact with me

P3
Concerned that my reputation among my friends has declined

P4
Concerned that my post was not appealing enough for them to want to Like or Retweet it or even comment on it

P5
Concerned with my profile being less active so that others lose interest

P6
Concerned that if people live outside my city or country they may not interact with me

Context2: When I am unable to interact or connect to social media as I wish, I may be:

P7
Concerned with missing a certain post because of the large number of posts or tweets

P8
Concerned with the difficulty of reaching the posts or tweets I need because of the large number of posts or tweets

P9
Concerned with locating an important message because I have messages from many different social media users

P10
Concerned with responding the most important messages received from different social media users

P11 Concerned that other people's posts may be removed or disappear; e.g. stories on Snapchat, urgent messages or news feeds that disappear after a period of time

P12 Concerned that I have to respond immediately to messages

P13 Concerned that my friends feel they are ignored

P14 Concerned with missing people who do not often post messages on social media so that I may not find them when I come back online

P15 Concerned with missing being involved in current discussions that may be interpreted to mean that I do not like to participate in friends' discussions

P16 Concerned I may miss supporting or defending friends, teams or opinions on Facebook, WhatsApp, Snapchat, Twitter, Instagram or others social media platforms

P17 Concerned at the inability to talk regarding missing online discussions when my friends are gathered at school, restaurants, coffee shops, home, etc.

P18 Concerned with the need to increase my activity on social media to keep or increase my followers

P19 Concerned with the need to update my profile frequently to keep or increase my followers

P20 Concerned at missing posts from a particular person (e.g. celebrities)

Context3: When I am unwilling to engage in social interaction (e.g. group chat), I may be

P21 Concerned about missing unexpected requests from group members

P22 Concerned about losing the benefits of being in the online group (e.g. plans for a party, assignment information)

P23 Concerned that group members will not respond to me in future

P24 Concerned that my friends feel they have been ignored

P25 Concerned about damaging relationships and reputations with others

P26 Concerned that I will not be involved in future discussions in online groups

Context4: When I keep checking or feel a need to engage in continuous untimed interactions, I may be:

P27
Concerned about reassuring friends I am interested in their conversation when I really want to stop the conversation

P28
Concerned about not harming my self-image when I really want to stop the conversation

P29
Concerned about not showing empathy when I really want to stop the conversation

P30
Concerned about not hurting other people's feelings (e.g. affect others' self-esteem) when I really want to stop the conversation

P31
Concerned about missing my social relationships

P32
Concerned about the need to reply to people who comment on my posts or tweets

P33
Concerned about the need to value people who comment on my posts or tweets

P34
Concerned about the need to delete a post if negative comments are made

P35
Concerned about doing something wrong before knowing others' impressions (making wrong decisions) when I asked them about something

P36
Concerned about the need to reply immediately

P37
Concerned about not people feeling ignored so that I feel the need to reply immediately

P38
Concerned about information needed by the sender when I ask them to do something to help me

P39
Concerned about missing valuable opportunities (e.g. advertisements or job opportunities)

P40
Concerned about missing unexpected requests from friends/group members

P41
Concerned about missing out on what others are doing

Context5: When I expect an online social gathering, I may be:

P42
Concerned about missing live chats

P43
Concerned about being unaware of whether or not people are available on social media

P44
Concerned about whether or not my name is mentioned in online groups

P45
Concerned about losing influence among my friends

BOOKLET 2:

List of FoMO countermeasures

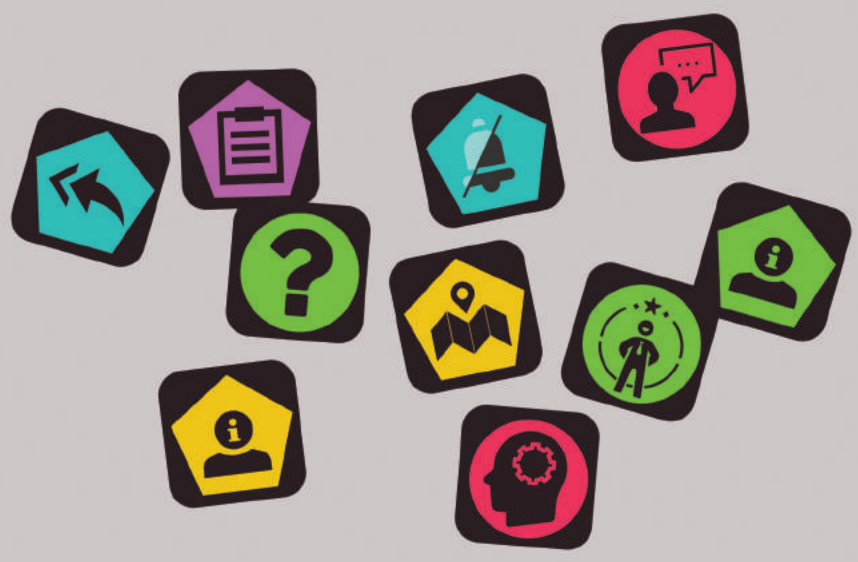

This booklet gives you variety countermeasures that can manage different kinds of FoMO. Based on your selection from booklet 1 select the suitable counter-measures for you in order to minimise your FoMO. for example, if you select P1 from booklet 1 you can find P1 in this document with the name of your FoMO and suggested countermeasures that may help you to manage your FoMO.

Please select the suitable countermeasure and then remove the sticker based on your selection from the stickers sheet.

IMPORTANT NOTES:

Some technical countermeasures have not existed yet on social media, but you can use an alternative option or socio countermeasures.

Fear of missing the ability to be interesting (P4, P5, P6)

Recommended technical countermeasures

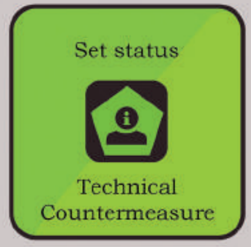

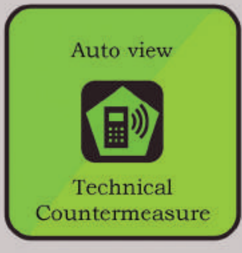

Set status e.g. you would like certain contacts to set their status in advance to show you whether they are online or available to interact or not.

Social media shows you who viewed your post, e.g. Snapchat provides this feature. You can 1. Go to your Profile on snapchat 2. Tap 'My Story' to view it 3. Tap eyeball to see who watched your story

Recommended socio-countermeasures - if you would like to use socio countermeasures, please go to page 3

Recommended socio-countermeasures for P1-P6

Practice Self-talk

Socio-Countermeasure

Try to **practice self-talk,** you can say: I do not expect interaction from others when I post on social media

Improve Self-esteem

Socio-Countermeasure

Try to improve your **self-esteem** by saying: I am not the only one who does not receive the interaction they expect

Set status

Technical Countermeasure

Try to **manage your expectation** by: posting on social media without expecting interaction from others OR interact with others without expecting reciprocal interactions

Controlling anxiety

Socio-Countermeasure

Try to **control your anxiety** by: distracting yourself by engaging in offline activities such as making coffee, cleaning the house, talking to the person next to you, walking around your house etc.

Ask questions

Socio-Countermeasure

Try to **ask yourself:** what you would say to friend who faced a similar situation

Pre-posting thinking

Socio-Countermeasure

Manage the post by using **pre-posting thinking** (See Think before post instructions on page 22)

Context 2: FoMO when unable to interact or connect as wished

EXEMPLAR CASE:

You are in a formal meeting and unable to check your social media even when you receive notifications. Also, you had a 5 minute coffee break but it wasn't enough to check all notifications and respond. Thus, you may experience the following FoMO but could practice the following techniques to reduce your fear:

KINDS OF FOMO

- Fear of missing information due to a large volume of information (P7, P8)

Recommended technical countermeasures

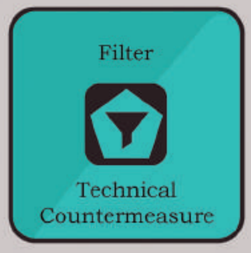

Filter, set your filter by classifying messages and notifications according to the topic and contacts involved that are interested to you, so that you can easily estimate their subject and importance, e.g. see instruction A (page 23) and B (page 24)

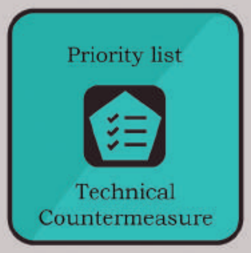

Priority list, set levels of importance and relevance to topics, contacts, etc that are interested to you, so that you are able to prioritize better.

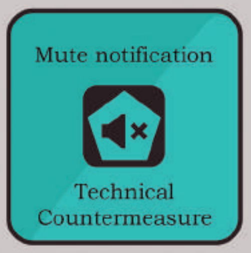

Alternative option, mute notifications from contacts or groups that are not high importance.

Recommended socio-countermeasures - if you would like to use socio countermeasures, please go to page 9

Fear of missing the ability to deal with different social media (P9, P10)

Recommended technical countermeasures

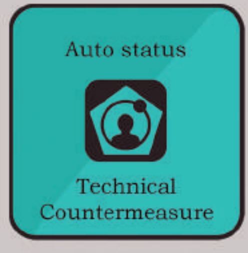

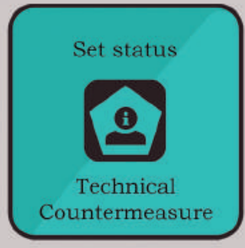

Auto-status, Facebook sets you as busy automatically when you are using WhatsApp. Alternatively, you can use the next technique.

Set status, set a status manually on a certain social media profile that you are busy on another conversation, e.g set you are busy on WhatsApp when you are using Facebook

Recommended socio-countermeasures - if you would like to use socio countermeasures, please go to page 9

Fear of missing temporally available information; stories, urgent messages or news feeds etc. that disappear after a while (P11)

Recommended technical countermeasures

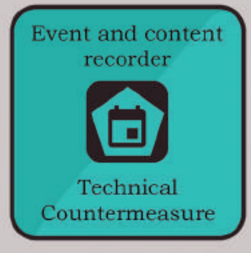

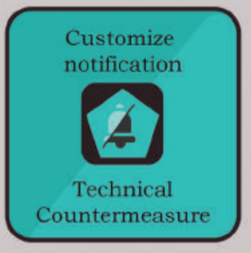

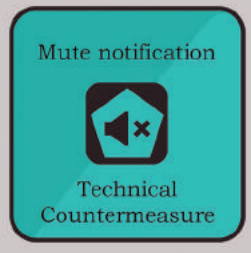

Event and content recorder, e.g. having an offline version downloaded so you can still see such temporarily available content when you have time.

Diverse notification, e.g. set different tones and alerts styles for such temporarily available information and when content is about to expire, etc, e.g. see instruction C (page 24)

Alternative option, mute notifications from contacts or groups that their stories are not

Recommended socio-countermeasures - if you would like to use socio countermeasures, please go to page 9

➡ Fear of missing a timely interaction; e.g. responding to friends' messages to accept an invite to a dinner party (P12, P13, P14)

Recommended technical countermeasures

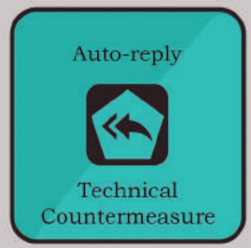

Auto-reply set some form of auto-reply that sends an automatic response to messages and informs your messaging contacts that you cannot respond immediately. E.g., you can set up auto-reply message on your email

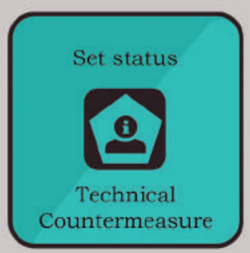

Set status, set social media status on your profile in advance saying that you are in a formal meeting or unavailable so they know in advance about your situation

Recommended socio-countermeasures - if you would like to use socio countermeasures, please go to page 9

Fear of missing participating in popular interactions (P15,P16,P17)

Recommended technical countermeasures

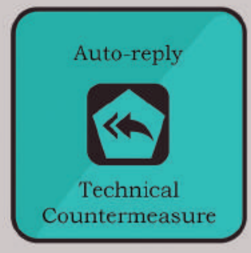

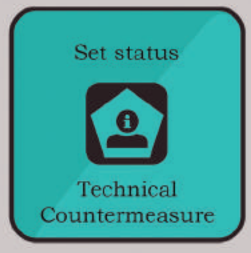

Auto-reply set some form of auto-reply that sends an automatic response to messages and informs your messaging contacts that you cannot respond immediately. E.g., you can set up auto-reply message on your email

Set status, e.g. you set a social media status in advance that shows your contacts and followers when you are able or unable to post and communicate online.

Recommended socio-countermeasures - if you would like to use socio countermeasures, please go to page 9

Fear of missing the ability to keep followers (P18, P19)

Recommended technical countermeasures

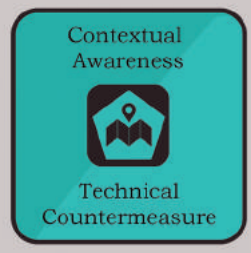

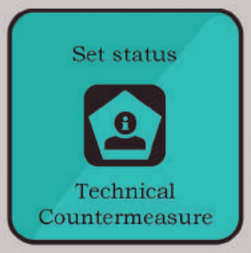

Contextual Awareness, allow a certain social media to access your calendar and your location on smart device that in order to updates your current situation, for example, busy or away, driving or current local time, etc. As example, see instruction F (page 25)

Set status, e.g. you set a social media status in advance that shows your contacts and followers when you are able or unable to post and communicate online.

Recommended socio-countermeasures - if you would like to use socio countermeasures, please go to page 9

Fear of missing information/events due to multi-following (P20)

Recommended technical countermeasures

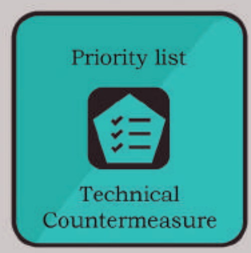

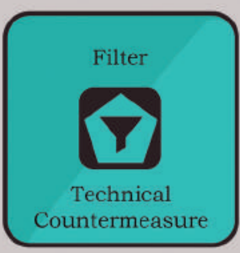

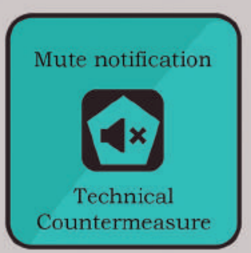

Priority list, set levels of importance and relevance to topics, contacts, etc that are interested to you, so that you are able to prioritize what you follow and connect to.

Filter, set a filter by classifying what followings are interested to you, e.g. see instruction A (page 23) and B (page 24)

Alternative option, mute notifications from followings that you are not highly interested to their post.

Recommended socio-countermeasures - if you would like to use socio countermeasures, please go to page 9

Recommended socio-countermeasures for P7-P20

Managing expectations

Socio-Countermeasure

Try to **manage your expectation** by: expecting to miss information

Keep informed

Socio-Countermeasure

Try to: **keep your contacts informed** about your current situation

Practice Self-talk

Socio-Countermeasure

Try to **practice self-talk,** you can say: I do not need to check all things on social media; I do not need to know everything about my contacts; or I do not need to check each notification immediately

Improve Self-esteem

Socio-Countermeasure

Try to **improve your self-esteem** by saying: my popularity is not measured by being active on social media and immediately response to messag-

Manage impulsive behaviour

Socio-Countermeasure

Try to **manage your impulsive behaviour** by: not reacting to each notification

Manage impulsive behaviour

Socio-Countermeasure

Try to **manage your impulsive behaviour** by: practicing not checking your social media for a period of time.

Context 3: FoMO when unwilling to engage in social interaction

EXEMPLAR CASE:

Your colleagues are discussing a topic that is of interest to you; e.g. sport or music events in an online group. You are receiving notifications but you do not engage and check because you are tired or not in the mood to do so. Thus, you may:

KINDS OF FOMO

- Fear of missing valuable information (P21, P22)

Recommended technical countermeasures

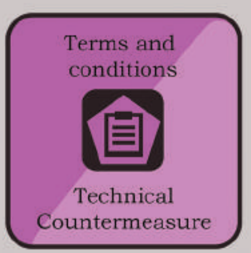

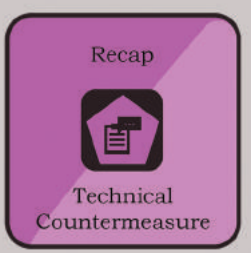

Terms and conditions, group members of WhatsApp or Facebook collectively agree terms and conditions for the group, e.g. times for posting, tolerating lack of responses, topics of interest, etc. **Alternative option** On WhatsApp Facebook, twitters etc. ask your group members to set terms and condition for the group on group description section

Recap, select recap for a certain social media group and then you get a summary and highlights of what is discussed in the group. As example for Recap, Snapchat gives you a recap monthely about your stories. Just Just open the Snapchat app and tap on the Memories icon as you normally would. Your month End Story will appear under the Snaps tab at the top of the screen

Recommended socio-countermeasures - if you would like to use socio countermeasures, please go to page 12

Fear of missing the ability to defend your popularity (P23, P24, P25, P26)

Recommended technical countermeasures

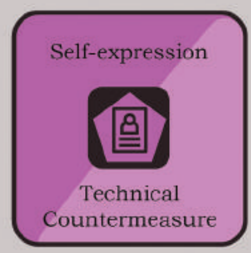

Self-expression, set details about yourself in advance such as your topic of interest, time of interactions and the style of your responsiveness, etc. **Alternative option**, set details about yourself in advance such as your topic of interest, time of interactions and the style of your responsiveness, etc. on your profile on social media

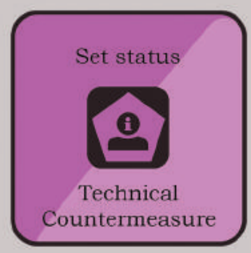

Set status, set Facebook or WhatsApp status in advance to inform other contacts such as your current mood or time availability in order for them not to feel ignored if you do not.

Recommended socio-countermeasures - if you would like to use socio countermeasures, please go to page 12

Recommended socio-countermeasures for P21-P26

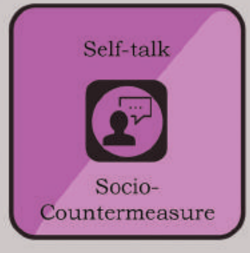

Try to **practice self-talk,** you can say:

My popularity does not depend on online participation,

I do not engage in each online group interaction

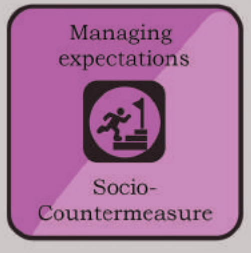

Try to **manage your expectations**:

by making your online group members informed about your current situation, mood

Try to manage your expectations: by expecting to miss information on social media

Context 4: FoMO when having to or feeling a need to engage in continuous and untimed interactions

EXEMPLAR CASE:

You are having a conversation on social media with a close friend. You want to stop the conversation but are unable to because you may:

KINDS OF FOMO

- Fear of missing empathy and leaving a good impression (P27, P28, P29,P30,P31)

Recommended technical countermeasures

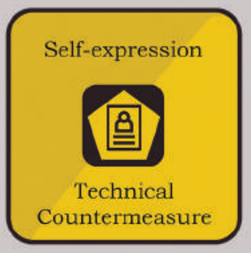

Self-expression, identify details about yourself in advance to your contacts before they interact with you such as your topic of interest, the maximum time for you conversations and the style of your responsiveness, etc. **Alternative option** identify details about yourself in advance to your contacts before they interact with you such as your topic of interest, the maximum time for you con versations and the style of your responsiveness, etc. on your profile on social media

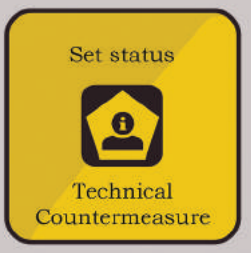

Set status, on your social media in advance to your contacts such as your other activities and the amount of time available to stay online,etc

Recommended socio-countermeasures - if you would like to use socio countermeasures, please go to page 18

EXEMPLAR CASE:

You posted a video on social media, e.g. Instagram, and you found it difficult to stop checking social media when you are doing some other activities (driving, physical exercise, etc.). You want to stop checking but you may

KINDS OF FOMO

➜ Fear of missing the opportunity to know others' impressions (P32, P33, P34, P35)

Recommended technical countermeasures

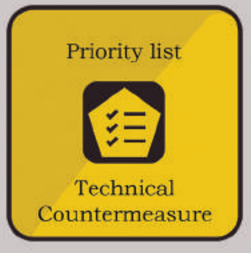

Priority list, set the level of importance of contacts that you would like to know their reactions to your posts.

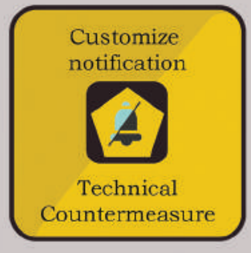

Diverse notifications, set different tone for specific contacts or reactions for specific posts, **Alternative option** Customize tone for your favourite contacts. As example, see instruction C (page 24)

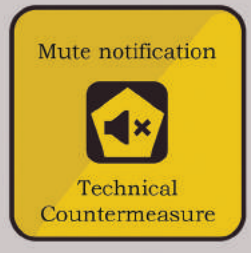

Alternative option, Mute notification from unwanted contacts or groups. As example, see instruction D (page 24)

Recommended socio-countermeasures - if you would like to use socio countermeasures, please go to page 18

Fear of losing popularity (P36, P37)

Recommended technical countermeasures

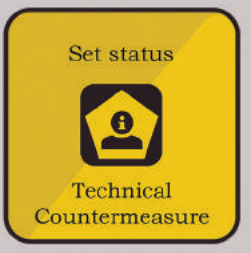

Set status, set social media status on your profile which can be seen in advance so that you say that you are currently busy

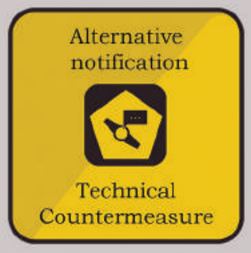

Alternative notification, set up SMS or a vibration notification from a certain social media on your smartwatch if you have an urgent email from some contacts, e.g. see instruction E (page 25)

Recommended socio-countermeasures - if you would like to use socio countermeasures, please go to page 18

Fear of missing spontaneous responses (P38)

Recommended technical countermeasures

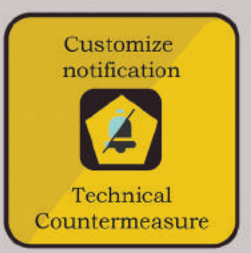

Diverse notification, set a different tone for contacts that you are waiting message from them, **Alternative options:** Customize tone for your favourite contacts. As example, see instruction C (page 24)

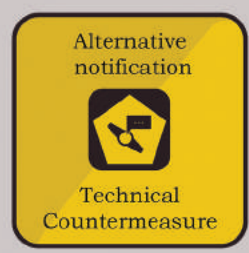

Alternative notification set up SMS or a vibration notification from a certain social media on your smartwatch if you have an urgent email from some contacts, **Alternative option**: Set up SMS notification on Facebook, see instruction E (page 25)

Recommended socio-countermeasures - if you would like to use socio countermeasures, please go to page 18

EXEMPLAR CASE:

On a sales season or a discount day such as Boxing day or Black Friday, You are sitting with your family or friends while thinking of buying something. You start to check brand sites and their apps and your shopping groups because you may

KINDS OF FOMO

Fear of missing a valuable opportunity (P39)

Recommended technical countermeasures

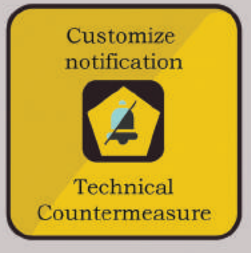

Diverse notifications, set different tone for specific brand, discount type and product. **Alternative option:** Customize tone for your favourite contacts. As example, see instruction C (page 24)

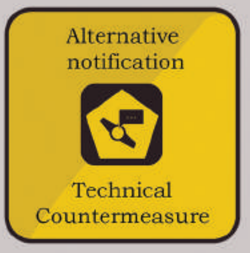

Alternative notification, set up SMS or a vibration notification from a certain social media on your smartwatch if there is a major discount for a specific brand. **Alternative option:** Set up SMS notification on Facebook, see instruction E (page 25) for instructions

Recommended socio-countermeasures - if you would like to use socio countermeasures, please go to page 18

Fear of missing a sense of relatedness (P40, P41)

Recommended technical countermeasures

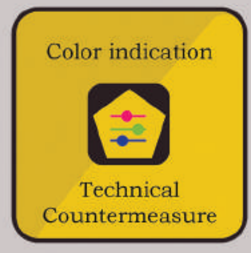

Color indication set a specific color in the alert light or the notification icon to distinguish specific information coming from this group.

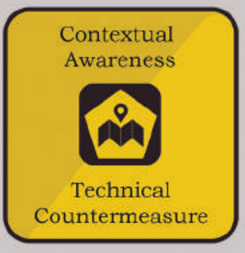

Contextual Awareness, allow a certain social media to access your calendar and your location on smart device that in order to updates your current situation, for example, busy or away, driving or current local time, etc. As example, see instruction F (page 25)

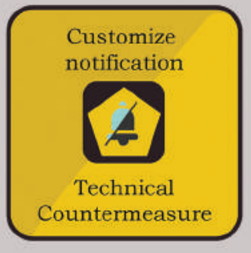

Alternative option: Customize tone for your favorite contacts. As example, see instruction C (page 24)

Recommended socio-countermeasures - if you would like to use socio countermeasures, please go to page 18

Recommended socio-countermeasures for P27-P41

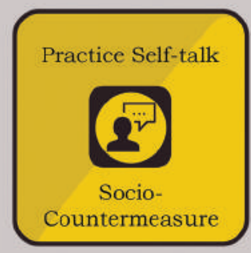

Try to **practice self-talk,** you can say:

I do not need to spend a long time on conversations because I have task to do; I have to sleep early etc.

my popularity does not depend on an immediate response to the message

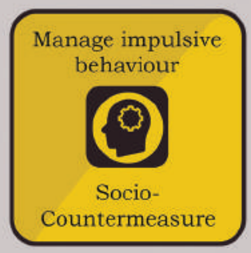

Try to **control your compulsive feelings by:** distracting yourself by

- Doing something that you enjoy
- Occupying yourself with work instead
- Keeping yourself busy
- Calling to mind positive images instead
- Thinking pleasant thoughts instead

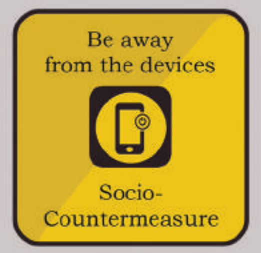

Try to **be away** from the phone or computer

Context 5: FoMO when an online social gathering is expected

EXEMPLAR CASE:

When you expect an informal online social gathering (e.g. group chat on Facebook or WhatsApp) if there is a possibility that you cannot connect as expected for the whole session time or part of it, you may:

KINDS OF FOMO

Fear of missing the opportunity to attend an online event (P42)

Recommended technical countermeasures

Calendar event reminder
Technical Countermeasure

Recap
Technical Countermeasure

Calendar event reminder, set a time and date of a specific event.

Recap e.g. you wish if there is a recap feature in social media that gives you a summary of what has been discussed in live chat, As example for Snapchat gives you a recap monthely about your stories. Just Just open the Snapchat app and tap on the Memories icon as you normally would. Your month End Story will appear under the Snaps tab at the top of the screen

Recommended socio-countermeasures - if you would like to use socio countermeasures, please go to page 21

Fear of missing a sense of relatedness (P43, P44)

Recommended technical countermeasures

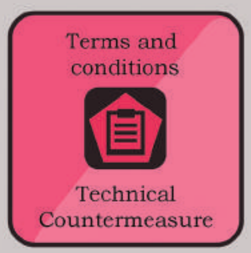

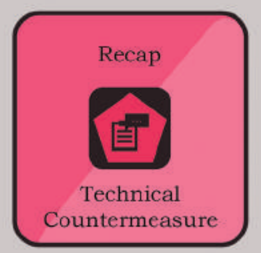

Terms and conditions, group members of WhatsApp or Facebook collectively agree terms and conditions for the group, e.g. times for posting, tolerating lack of responses, topics of interest, etc. **Alternative option** , On WhatsApp Facebook, twitters etc. ask your group members to set terms and condition for the group on group description section

Color indication, set a specific color in the alert light or the notification icon to distinguish specific information coming from this group.

Recommended socio-countermeasures - if you would like to use socio countermeasures, please go to page 21

Fear of missing the ability to be popular (P45)

Recommended technical countermeasures

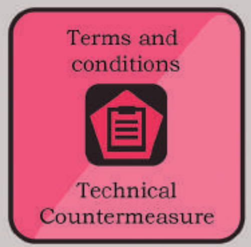

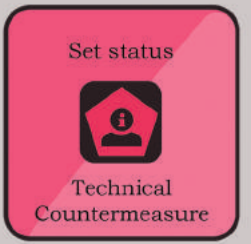

Terms and conditions,group members of WhatsApp or Facebook collectively agree terms and conditions for the group, e.g. times for posting, tolerating lack of responses, topics of interest, etc. **Alternative option** , On WhatsApp Facebook, twitters etc. ask your group members to set terms and condition for the group on group description section

Set status, e.g. you set a status on your social media in advance to your contact such as your other activities and the amount of time available to stay online, etc.

Recommended socio-countermeasures for P42-P45

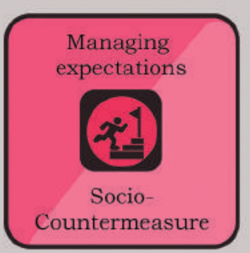

Try to manage your expectation by: expecting to miss it
-Try to: keep your contacts informed about your current situation

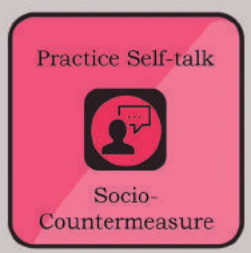

Try to practice self-talk, you can say: I do not need to check all things on social media; I do not need to know everything about my contacts; or I do not need to check each notification immediately

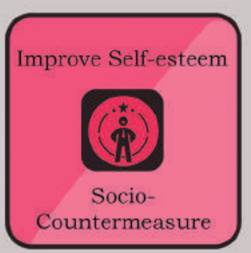

Try to improve your self-esteem by saying: my popularity is not measured by being active on social media and immediately responding to messages

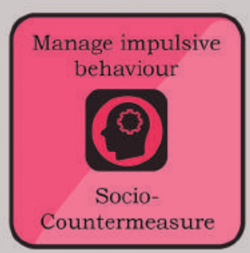

Try to manage your impulsive behaviour by:

not reacting to each notification

practicing not checking your social media for a period of time

Think before post

To think before post or send please use the following checklist:

Does the post or message have value to the audience?

YES NO → Choose a different topic

Does the post's content fit the audience's interests?

YES NO → Choose a different audience

Is this the best format and language for the post?
For example:
- Emoji may not be understood by others
- Text language may not be understood by others, e.g., BRB, BTW,SBY,HAND, etc

YES NO → Adjust your format and language,

Is this the best time for a certain audience?

YES NO → Schedule for another time by looking to the audience's availability

If your answers are "yes" for all questions, it may be good to post or send but bear in mind the various expectations:

- Expect few interactions
- Expect No interactions
- Expect No immediate response
- Expect not all are interested
- Expect others are on leave
- Expect others may need time to process
- Expect the algorithm does not show your post to them
- Expect others could not connect to the internet
- If you do not receive interactions from someone who is online you can expect they may have an urgent or business conversation
- If you do not receive interactions from someone who is online you can expect they may not be prepared to answer
- If you do not receive interactions from someone who is online you can expect they may be busy in reading a business message

Instruction A

Search from Twitter

If you search from the web ,please follow the instruction below

1. Enter your search query into the **search box** at the top of the page.
2. Your results will show a combination of Tweets, photos, accounts, and more.
3. Filter your results by clicking **Top, Latest, People, Photos, or Videos** (located at the top of your search results).
4. Click the **Search filters** options to filter your results From anyone or **People you follow**, and **Anywhere** or **Near you**

If you search from the IOS, please follow the instruction below

1. Tap the **Explore** tab
2. At the top of the page, enter your search into the search box and tap Search.
3. Your results will show a combination of Tweets, photos, accounts, and more.
4. Filter your results by tapping **Top, Latest, People, Photos, Videos, or News, or Periscopes** (located at the top of your search results)
5. Tap the filter icon in the search bar to refine your results according to **All people or People you follow, and Everywhere or Near you.**

If you search from the Android, please follow the instruction below

1. Tap the **Explore** tab
2. Enter your search into the search box and tap Search.
3. Your results will show a combination of Tweets, photos, accounts, and more.
4. Filter your results by tapping **Top, Latest, People, Photos, Videos, or News, or Periscopes** (located at the top of your search results).
5. Tap the filter icon in the search bar to refine your results according to **From anyone or People you follow, and Anywhere or Near you.**

Instruction B

To adjust your Facebook News Feed preferences

Your News Feed preferences help you control what you see on your News Feed. To view your News Feed preferences:

1. Click ▼ in the top right corner of any Facebook page (example: your home page).
2. Select News Feed Preferences.
3. Click Prioritize who to see first to make posts from people or Pages appear at the top of your News Feed

Instruction C

To set special notification

1. Go to the group or contacts in WhatsApp, then tap the subject of the group or the contact.
2. Tap the custom tone.
3. Select Custom notifications.
4. Click save on the top right.

Instruction D

To control comment's notification for your post

To set notification about what you get notifications about:

1. Click ▼ at the top-right corner and select Settings.
2. Click Notifications on the left.
3. Click comments to adjust how you get notifications

Note: please turn off the rest of notifications in order to just notify regarding the comments

Instruction E

To set up Facebook texts

1. Click at the top right of any Facebook page and select Settings.
2. Click Mobile.
3. If you haven't added your mobile phone number to your account, click Add a Phone and follow the steps. If you've already added a mobile phone number to your account, click Activate Text Messaging.

Once you set up Facebook texts, you can receive texts (SMS) from Facebook and update Facebook by sending a text. . However if you do not receive SMS follow the following steps

1. Click ▼ at the top-right corner and select Settings.
2. Click Notifications on the left.
3. Click comments to adjust how you get notifications
4. Select SMS

Note : Please turn off the rest of notifications in order to just notify regarding the comments

Instruction F

Set a Status at Location on Snapchat

You can set a Status at locations on the Snap Map. Your tatus updates your Bitmoji on the Map with a text bubble that says where you are.

To Set a Status at Location on Snapchat:
1. Swipe down on the Camera screen to open the Map
2. Tap 'Status' at the bottom
3. Tap a location
4. Tap a Bitmoji for your new Status and tap 'Set Status'

Your Status lasts four hours, or until you leave that location or set a new Status. You can check out who's viewed your Statuses when you tap the 'Status' button.

BOOKLET 3

Relapse prevention

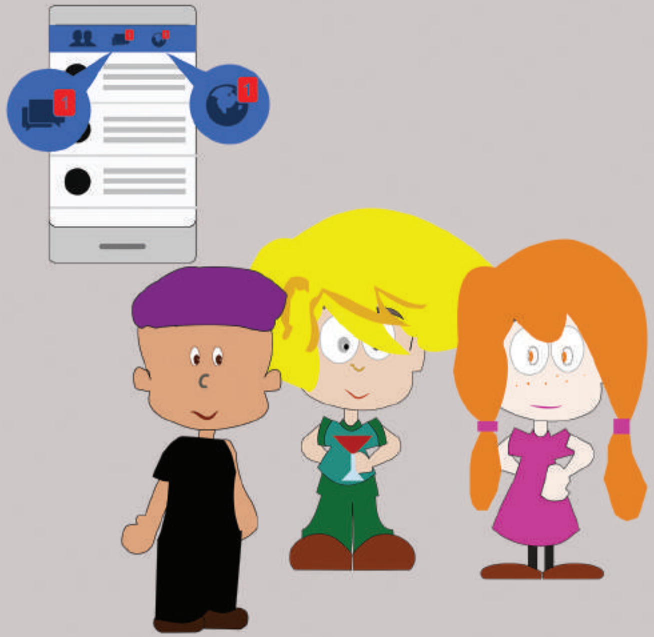

WHAT IS RELAPSE?

Relapse “refers to a breakdown or failure in a person's attempt to change or modify any target behavior”

WHAT IS RELAPSE PREVENTION?

Relapse Prevention (RP) is a self-control technique designed to help individuals who are trying to change their behavior on how to anticipate and cope with the problem of relapse.

IMPORTANT POINTS, PLEASE READ THEM:

1. If you manage your FoMO for a while and then begin to experience again, you relapsed.
2. Relapse happens because people may stop using the FoMO reduction method.
3. Relapse can be avoided by willpower and self-discipline alone.

RELAPSE PREVENTION PLAN

Coping skills: please list activities or skills you enjoy that can get your mind off of using

TO PREVENT RELAPSE:

1. Please do one or more of activities or skills that you listed on the relap prevention plan,

OR

2. Please do hobbies such as a reading book, walking, exercise, club membership, etc.

OR

3. Practice positive self-talk, e.g.

YOU CAN SAY

“Even though I am frustrated with not being connected or checking, I am open to the possibility I can find a calm space within myself.”

“Even though I am anxious about not being connected or checking, I am open to the possibility I will feel better soon”

“Even though I am anxious about not received responses from others I know they did not do this on purpose”. thin myself.”

“Even though I am anxious about not being connected or checking, I am open to the possibility I will feel better soon ”

.

“Even though I am anxious about not received responses from others I know they did not do this on purpose”.

BOOKLET 4

Empowered refusal and Self-talk

Please determine which is the challenges that prevent you from managing your FoMO?

Peer pressure	Putting others needs above your own	Technical issues
↓	↓	↓
Try to apply Empowered refusal with goal setting	Try to apply Empowered refusal with goal setting	Try to apply Tiny habit model
↓	↓	↓
See 4.a	See 4.b	See 4.c

4.a. Empowered refusal with goal setting

You can phrase your goal as a refusal statement by using *I do not need.* Please see examples below

Example:

- *I do not need to participate in the group when I am on the lecture*
- *I do not need to reply when I am driving*

4.b. Self-talk sentences with goal setting

You can phrase your goal by using self-talk technique. Please see the instruction below:

- Try to phrase your goal in terms of a statement as a question, e.g. will you need to read all posts on twitter?
- Try to refer to yourself by using second-person pronouns, e.g. you need to express your type of interactions on Facebook

4.c. Tiny Habits Model

Instruction to use Tiny Habits Model:
This sheet is a way to match up habits that you already have in your life with new goals you have for yourself and the habits you want to begin to have in order to reduce your FoMO. Think of some new small habits you would want to have in your life, e.g. set status for short period of time. Next, think of some habits you already have in your life that could be a trigger for the new goal. Every time you perform the habit, it will remind you to perform the action you set as your goal.

They should happen at the same time every day and the things you do daily. Also, if the goal is something you want to do more than once a day, make sure to think of existing habits that you do more than once per day. After you finish listing out your thoughts, take time to match up which goal is most important with the habit that works best as a trigger. Remember to start small and simple, it will help you be the most successful.

Please see example below as an assistant

**Existing Habits:**	**New Tiny Goals:**
After I sat on my desk	I will set status as busy
After I have my dinner	I will participate in Facebook group

**Existing Habits:**	**New Tiny Goals:**
